# Microbiome modeling: a beginner's guide

**DOI:** 10.3389/fmicb.2024.1368377

**Published:** 2024-06-19

**Authors:** Emanuel Lange, Lena Kranert, Jacob Krüger, Dirk Benndorf, Robert Heyer

**Affiliations:** ^1^Multidimensional Omics Data Analysis, Department for Bioanalytics, Leibniz-Institut für Analytische Wissenschaften - ISAS - e.V., Dortmund, Germany; ^2^Graduate School Digital Infrastructure for the Life Sciences, Bielefeld Institute for Bioinformatics Infrastructure (BIBI), Faculty of Technology, Bielefeld University, Bielefeld, Germany; ^3^Institute for Automation Engineering, Otto von Guericke University Magdeburg, Magdeburg, Germany; ^4^Engineering of Software-Intensive Systems, Department of Mathematics and Computer Science, Eindhoven University of Technology, Eindhoven, Netherlands; ^5^Applied Biosciences and Bioprocess Engineering, Anhalt University of Applied Sciences, Köthen, Germany; ^6^Multidimensional Omics Data Analysis, Faculty of Technology, Bielefeld University, Bielefeld, Germany

**Keywords:** systems microbiology, microbial ecology, omics data integration, human microbiome, genome-scale modeling, constraint-based modeling, computational biology, bioinformatics

## Abstract

Microbiomes, comprised of diverse microbial species and viruses, play pivotal roles in human health, environmental processes, and biotechnological applications and interact with each other, their environment, and hosts via ecological interactions. Our understanding of microbiomes is still limited and hampered by their complexity. A concept improving this understanding is systems biology, which focuses on the holistic description of biological systems utilizing experimental and computational methods. An important set of such experimental methods are metaomics methods which analyze microbiomes and output lists of molecular features. These lists of data are integrated, interpreted, and compiled into computational microbiome models, to predict, optimize, and control microbiome behavior. There exists a gap in understanding between microbiologists and modelers/bioinformaticians, stemming from a lack of interdisciplinary knowledge. This knowledge gap hinders the establishment of computational models in microbiome analysis. This review aims to bridge this gap and is tailored for microbiologists, researchers new to microbiome modeling, and bioinformaticians. To achieve this goal, it provides an interdisciplinary overview of microbiome modeling, starting with fundamental knowledge of microbiomes, metaomics methods, common modeling formalisms, and how models facilitate microbiome control. It concludes with guidelines and repositories for modeling. Each section provides entry-level information, example applications, and important references, serving as a valuable resource for comprehending and navigating the complex landscape of microbiome research and modeling.

## Graphical Abstract

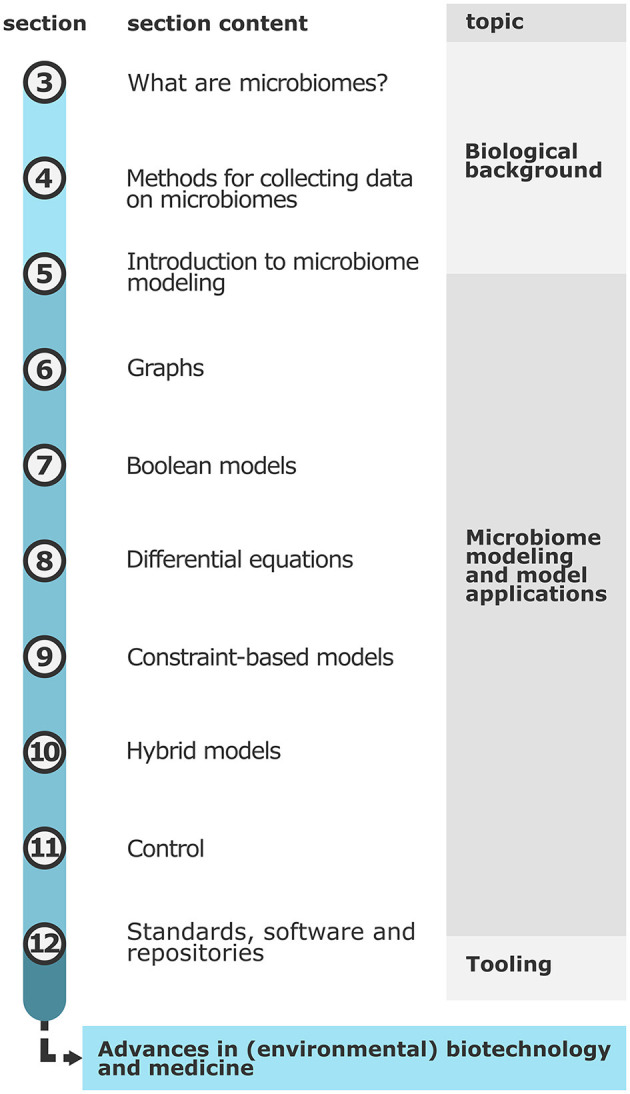

## 1 Introduction

Most habitats on earth are populated by microbiomes consisting of various microbial species and viruses.^1^ Due to their ubiquity and versatility, microbiomes are essential for human life, development, and health (Cani, 2018; Gilbert et al., 2018). The human microbiome can, for instance, increase cancer risk and progression by promoting local chronic inflammation, the release of free radicals, or the induction of pro-inflammatory cytokines (Helmink et al., 2019). The intestinal microbiomes of livestock ferment feed that is indigestible for humans. Products from livestock such as meat or milk are valuable protein sources but cause 30% of the global anthropogenic methane emission at the same time (Jackson et al., 2020). Similar microbiomes as in livestock degrade organic waste and renewables in anaerobic digesters to biogas, which can be used for the production of renewable electric energy. In Germany, electricity from biogas covered about 5.8% of the electricity demand^2^ and contributed 10% to the prevented greenhouse gas emissions in 2022.^3^ Lastly, microbiomes play a major role in nutrient cycling and are important for soil fertility and plant growth (Naylor et al., 2020). These examples demonstrate how important microbiomes are for human health, biotechnology, and the environment.

Despite their importance, member species of most natural microbiomes are unknown (Amann et al., 1995; Wade, 2002) and their behavior is not fully understood (Gilbert et al., 2018). The reason for the lack of knowledge is the complexity of ecological interactions between microbiome members and their environment/hosts. Parts of the missing knowledge on microbiomes can be uncovered by metaomics methods. These analytical methods identify and quantify genes, transcripts, proteins, and metabolites in microbiomes (Qin et al., 2010; Aguiar-Pulido et al., 2016; Heyer et al., 2017) analyzing many samples and molecules in a relatively short time, thus branded as high throughput. Making sense of the high throughput of metaomics data requires bioinformatics for automated data integration and analysis (Henry et al., 2010; Heyer et al., 2017; Jünemann et al., 2017).

Metaomics data analysis results in mechanistic knowledge, which can be used to construct mathematical models of microbiomes (Faust and Raes, 2012; Tobalina et al., 2015; Machado et al., 2018; Aden et al., 2019; Marcelino et al., 2023). Model predictions can support or falsify hypotheses or complement data, advancing the understanding of microbiomes. Furthermore, model predictions can guide strategies to optimize and control the processes performed by microbiomes. For example, models can determine optimal conditions for producing chemical compounds (García-Jiménez et al., 2021), drug targets for growth inhibition of pathogens (Curran et al., 2020), or control the production of chemical compounds or biogas on-line (Xue et al., 2015; Espinel-Ríos et al., 2023a,b).

Although many reviews on microbiome modeling exist (Biggs et al., 2015; Kumar et al., 2019; García-Jiménez et al., 2021; van den Berg et al., 2022; Garza et al., 2023; Liu, 2023), they usually require background knowledge or do not mention the tools to get started with microbiome modeling. This review is intended to close this gap and explicitly targets beginners in microbiome modeling, offering a starting point for further exploration of the field. Therefore, the manuscript addresses the following aspects:

- First, the manuscript provides a concise background on the characteristics of microbiomes (Section 3) and metaomics methods to analyze them (Section 4).
- Second, general aspects of modeling (Section 5) and the most common modeling frameworks are explained (Sections 6 to 10). Each model section explains theoretical basics, important methods for model analysis, and provides examples of applications to microbiomes. Furthermore, references to important articles or reviews are provided, as well as lists of software to apply the corresponding model framework.
- Third, an introduction to strategies for controlling microbiomes and the contribution of microbiome models is given (Section 11).
- Fourth, important guidelines facilitating reusability and reproducibility of microbiome modeling are introduced (Section 12).

## 2 Methods

This review addresses microbiome characteristics, metaomics methods, microbiome modeling, and guidelines for improving the reuse of microbiome models. A Python script was used to retrieve an initial collection of papers from the respective fields. The script queries the PubMed API (Sayers, 2009), obtains a list of articles, and determines the most cited references across these articles (the used queries are listed in Table 1). The script was inspired by an available project (https://github.com/paulamartingonzalez/Targeted_Literature_Reviews_via_webscraping) and is available on our GitHub repository (https://github.com/voidsailor/targeted_literature_search, https://zenodo.org/doi/10.5281/zenodo.10402352).

**Table 1:** Pubmed queries.

**Script query**	**Date**	**Number of hits**	**Topics**
(microbiome) AND (microbial community)	November 7, 2023	4,465	Role and properties of microbiomes
(metaproteomics) OR (metagenomics) OR (metaomics)	November 7, 2023	4,200	Metaomics methods, metaproteomics, bioinformatic challenges
(computational model) AND [(metabolism) OR (regulation) OR (signaling)]	November 7, 2023	3,163	model types, modeling approaches applicable to metabolism and signaling
(biological network reconstruction) AND [(microbiome) OR (microbial community)]	November 7, 2023	6,531	Reconstruction of metabolic and signaling networks
(computational model) AND [(parameter estimation) OR (contextualization) OR (reduction)]	November 7, 2023	1,035	parameter estimation, context-specific models, reduction of model size
(computational modeling) AND [(microbiome) OR (microbial community)]	November 7, 2023	1,035	examples of prediction, optimization
(control algorithm) AND [(microbiome) OR (microbial community)]	November 7, 2023	4,313	microbiome control
(network modeling) AND (guidelines OR software OR repository)	November 7, 2023	1,671	FAIR, initiatives, standards, languages, software, repositories

The parameter for the initial number of papers was always set to 100. The most cited papers were extracted from the references of these initial 100 and ordered by node degree of the reference network. The best-fitting articles were selected for the respective topics, starting with the highest-ranked articles. The generated output files are in the Supplementary Table S1.

Further references were discovered from these primary articles or by subjecting interesting articles to the Connected-Papers web application.^4^

## 3 What are microbiomes?

**Figure 1:**
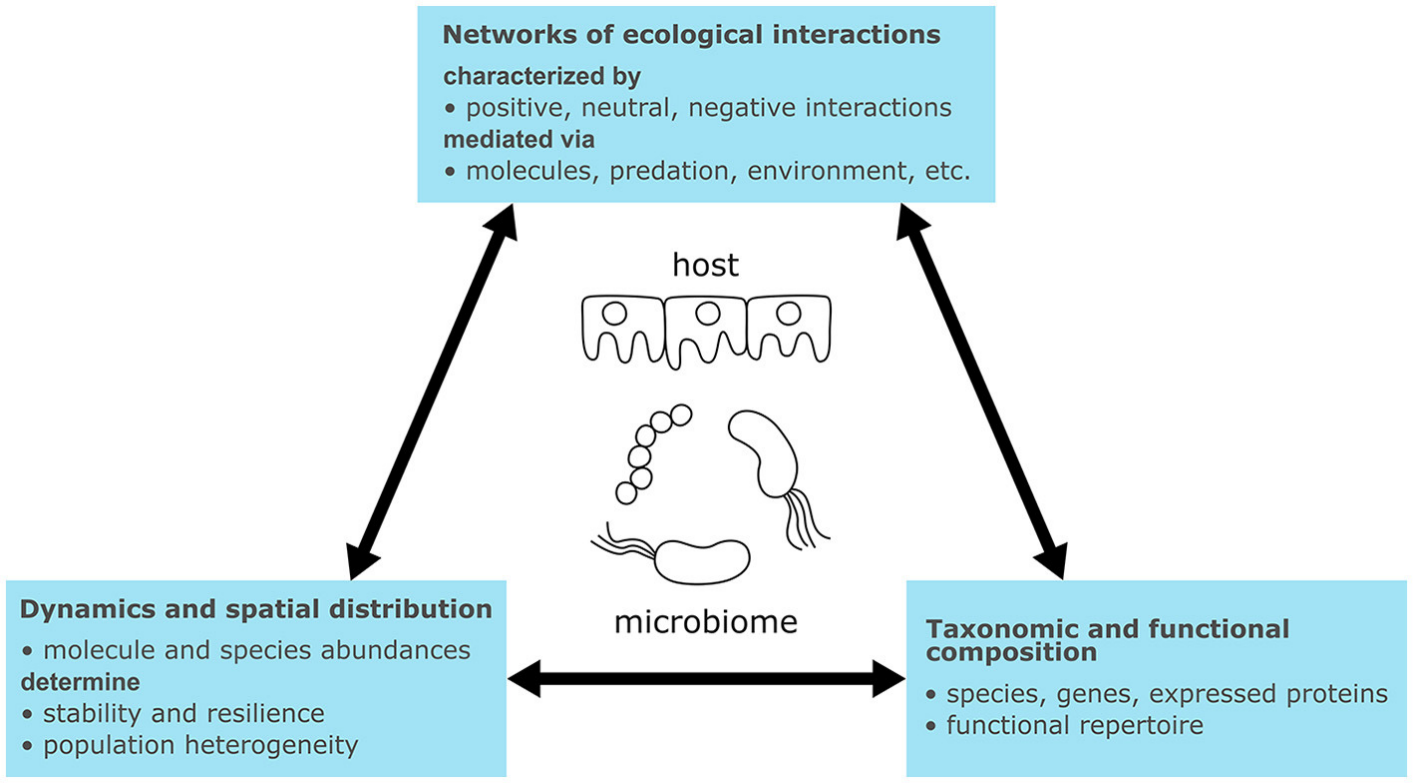
Characteristics of microbiomes with relevance for their general understanding and modeling.

Microbiomes are biological systems of heterogeneous communities of microorganisms living in the same habitat or host, engaging in non-linear and dynamic interactions (Figure 1). Microorganisms and host cells are driven by cellular metabolism, involving the uptake, conversion, and excretion of chemical compounds through networks of enzymatic reactions. These reactions generate energy and building blocks for cellular maintenance and growth (Berg et al., 2013a). Cellular signaling detects and processes external stimuli (e.g., pH, osmolarity, temperature, or signaling molecules). Cells receive these signals via membrane-bound or intracellular receptor proteins, which detect stimuli and transduce signals through cascades of sequentially activated proteins and small molecules (2^nd^ messengers) (Berg et al., 2013a). Terminal molecular signals induce cellular responses, such as changes in cellular shape (Huang et al., 2021), or activate gene expression through transcription factors (Berg et al., 2013d). Activated genes regulate metabolism and signaling by expressing regulatory RNAs, enzymes, and signaling proteins. Additionally, genes regulate other genes by expressing transcription factors forming gene regulatory networks. These networks encode biological programs that correspond to behaviors or phenotypes (Davidson and Levin, 2005; Berg et al., 2013b,c). The connection of molecular interactions forms feed-forward and feed-back loops determining dynamic system behaviors such as signal amplification or oscillation (Samaga and Klamt, 2013).

Ecological interactions between microbiome members and their hosts can be broadly categorized as mutualistic, neutral, or negative interactions (Berg et al., 2020) [for an overview on ecological interaction types see Fassarella et al. (2020) or García-Jiménez et al. (2021)]. For example, cross-feeding represents a positive (mutualistic) mechanism wherein organisms produce substrates for each other. Conversely, competition is a negative interaction wherein organisms compete for the same resource (García-Jiménez et al., 2021). The exchange of signaling molecules represents another type of interaction mediating processes such as quorum sensing. In quorum sensing, microorganisms respond with biofilm formation if the concentration of a signaling molecule exceeds a threshold, thereby enhancing the population's resilience to the environment (Solano et al., 2014). Other types of interactions are mediated by antimicrobial peptides or attractants (Quiza et al., 2015; Ma et al., 2022), phages (Federici et al., 2020), predation (Thakur and Geisen, 2019), or abiotic factors (Abdul Rahman et al., 2021). Microbial interactions can be pairwise, occurring between two species, but pairwise interactions can also be modulated by higher-order interactions with third species (Ludington, 2022).

Microbiomes contain hundreds to thousands of species spanning all domains of life (i.e., Archaea, Bacteria, Eukaryotes, and Viruses) and their taxonomic composition is usually unique to sample sites or hosts (Lozupone et al., 2012; Liu, 2023). Determining the taxonomic composition is of interest to identify the microbiome members that perform ecological interactions. Certain species can indicate specific biological processes; for instance, *Clostridium thermocellum* is capable of cellulose degradation in the biogas process (Heyer et al., 2015). However, while taxonomic profiles may vary considerably, functional profiles can remain similar (Lozupone et al., 2012). Therefore it is also informative to determine the functional repertoire encoded in genes and expressed in proteins.

Ultimately, the interactions connect members of the microbiome into an ecological network and determine its dynamics of species abundances (i.e., taxonomic composition) and concentrations of exchanged molecules (Liu, 2023). Like regulatory and signaling networks, feed-forward and feed-back loops can be found in microbiomes. For instance, cross-feeding results in coupling or positive feedback loops, while competitive interactions introduce negative feedback (Coyte et al., 2015). Such loops determine steady states of microbiomes, which correspond to equilibria between all interactions. Multiple steady states can exist for the same process, as observed for the taxonomic composition in lab-scale biogas plants (Kohrs et al., 2017). Ecological interactions also determine whether the steady states are resilient to perturbations (stable steady states) or not (unstable steady states) (Fassarella et al., 2020). For example, a high ratio of negative to positive interactions has been linked to increased resilience through negative feedback (Coyte et al., 2015) and resistance toward invasion of new species (Machado et al., 2021), while positive interactions such as cross-feeding may lead to more efficient substrate utilization through division of labor but lower resilience due to growth coupling (Coyte et al., 2015; Roell et al., 2019; Machado et al., 2021). Another aspect is functional redundancy, which is generally associated with higher resilience (Liu, 2023). The environment also has an impact on microbiome interactions and dynamics. For example, human microbiomes from different body sites differ in composition due to various physical conditions (e.g., the pH value) (The Human Microbiome Project Consortium, 2012). These conditions can exhibit their own dynamics, influenced by factors such as meal intake or the menstrual cycle (Liu, 2023).

Environmental conditions do not only vary macroscopically but also microscopically due to the spatial organization of cells. Microorganisms can live free-floating, as aggregates, or attached to surfaces in biofilms (Cai, 2020). Consequently, cellular density varies considerably depending on the environment (e.g., 10^6^ cells in 1 m^3^ air or 10^11^-10^12^ per mL in the colon) (Blum et al., 2019). The type of organization influences the mass transfer of molecules across the microbial population. Microorganisms at the surface of a biofilm can, for example, consume available oxygen completely and create anaerobic conditions inside the biofilm (Rani et al., 2007). Additionally, inter-individual variations can exist within the same population, giving rise to macroscopic effects (Kreft et al., 2017).

## 4 Collecting information on microbiome members

Cultivating and characterizing microbiome members is required to disentangle their roles within microbiomes. Moreover, cultivation experiments yield valuable data for microbiome modeling. Nonetheless, many species and microbiomes are difficult to grow in the lab, necessitating the analysis of microbiomes *in situ*. The following sections provide an overview of the challenges associated with cultivating individual species and microbiomes (Section 4.1), as well as metaomics methods for characterizing taxonomic and functional compositions of native microbiomes and their molecular repertoires (Section 4.2).

### 4.1 Cultivation and characterization of microorganisms

Most microbial species are still uncharacterized (Amann et al., 1995; Wade, 2002; Almeida et al., 2019; Pasolli et al., 2019). Out of the estimated 0.8–1.6 million prokaryotic species (based on operational taxonomic units) (Louca et al., 2019), about 0.7 million have sequenced genomes [NCBI, https://www.ncbi.nlm.nih.gov/genome/browse#!/prokaryotes/ (accessed April 24, 2024)], but less than 10% are available as isolates from the German Collection of Microorganisms and Cell Cultures [https://www.dsmz.de/ (accessed April 24, 2024), 26,766 bacterial and 634 archaeal strains].

Characterizing unknown microorganisms requires cultivation-based studies to determine the functions of their genes (Overmann et al., 2017). However, many species are difficult to grow in enriched or axenic cultures (i.e., single-species cultures), due to unknown nutritional requirements, or because they can only survive in synthrophies (Wade, 2002). Ongoing efforts optimize media and culture conditions for axenic cultures (Overmann et al., 2017). Furthermore, synthrophic species have been successfully grown and characterized in co-cultures with their interaction partners (Overmann et al., 2017). The resulting resources on characterized prokaryotic species are collected in databases such as BacDive (Reimer et al., 2021).

Growth experiments in axenic lab cultures are required to parameterize microbiome models (Section 5). Such cultures can provide enough material to determine cellular dry weight, macro-molecular biomass composition, ATP-maintenance coefficients, metabolic fluxes (Zamboni et al., 2009; Vos et al., 2016; Beck et al., 2018; Lachance et al., 2019) or analyze biomolecules by omics methods (Palazzotto and Weber, 2018). It is beneficial to plan experiments with modeling assumptions in mind. For example, constraint-based modeling (Section 9) assumes constant cellular metabolite concentrations and growth rates. Therefore, cultivation in continuously stirred tank processes is suitable to determine parameters for metabolic modeling, because process parameters remain constant (Winter and Krömer, 2013).

Lab cultures of reduced microbiomes (i.e., two to ten species) allow investigation of species interactions under controlled conditions. Reduced cultures are used to mimic the functional composition of more complex microbiomes, for example, biogas-producing microbiomes (Koch et al., 2016, 2019), or the human gut microbiome (Venturelli et al., 2018; Schäpe et al., 2019). It is also possible to inoculate lab cultures with samples from native microbiomes (Hanreich et al., 2013).

In many instances, microbiomes need to be analyzed in their native environments because native and lab-cultured microbiomes may differ in their phenotypes. Mesocosm experiments are a compromise between the native environment and controlled conditions. In such experiments, organisms are subjected to environments similar to their native environment, but specific conditions can be controlled (Lui et al., 2021; Petersen et al., 2023).

Microbiomes can furthermore be investigated using flow cytometry. Flow cytometry sorts and counts cells according to cellular features or chemical labels. Sorted cells can also be subjected to further (omics) analyses or cultivation (Props et al., 2016; Hatzenpichler et al., 2020). Lastly, microscopic observation gives clues about present species and is necessary to determine cellular morphology (e.g., shape, cell sizes, and spatial organization) (Xavier et al., 2007; Cesar and Huang, 2017).

### 4.2 Metaomics create inventory lists of microbiomes

Metaomics methods identify and quantify genes (metagenomics), transcripts (metatranscriptomics), proteins (metaproteomics), and metabolites (metabolomics) from complex or native microbiomes. The metaomics workflow generally begins with the extraction of molecules of interest which can be challenging due to complex sample matrices. Samples such as soil, sludge from wastewater treatment plants, or biogas plants, contain large amounts of impurities (e.g., minerals, humic substances) (Heyer et al., 2015; Starke et al., 2019). These impurities must be removed during sample preparation since they can disturb following workflow steps. Depending on the localization of molecules, cells need to be disrupted and any cellular processes that might alter the molecular profiles should be inhibited (Mashego et al., 2006; Bag et al., 2016; Bashiardes et al., 2016; Heyer et al., 2017). Subsequent purification steps aim to remove unwanted molecules (Thomas et al., 2012; Heyer et al., 2017). In metagenomics and metatranscriptomics, microbial RNA or DNA is sequenced, yielding sequence reads (Thomas et al., 2012; Bashiardes et al., 2016). In metaproteomics, proteins are denatured after purification, digested to peptides using trypsin, and subjected to liquid chromatography coupled to tandem mass spectrometry (LC-MS/MS), producing mass spectra (Heyer et al., 2017). In metabolomics, metabolites undergo analysis via mass spectrometry or nuclear magnetic resonance (NMR) analysis, resulting in mass or NMR spectra, respectively (Zhang et al., 2012). Finally, the raw data from each method undergo bioinformatic analyses (Thomas et al., 2012; Bashiardes et al., 2016; Heyer et al., 2017; Jünemann et al., 2017; Bauermeister et al., 2021), which extract information on the underlying ecological networks by identifying and quantifying measured molecules.

Important metagenomics methods are whole metagenome shotgun sequencing (WGS) and amplicon sequencing. WGS processes snippets of sequenced DNA (i.e., reads) to discern present taxonomies or functions along with their quantities (i.e., taxonomic and functional profiling). Reads can also be used for the *de novo* reconstruction of genomes (i.e., metagenome-assembled genomes, MAGs) of unknown organisms (Jünemann et al., 2017; Yang et al., 2021). However, MAGs can be incomplete or contain genes from different organisms. Taxonomy can be determined by marker genes or searches against databases containing known reference sequences (Jünemann et al., 2017), usually following the taxonomies assigned by the GTDB database (Parks et al., 2021). Functional annotations of genes can be obtained from reference databases or through homology searches against databases for functional ontologies or protein families, such as KEGG or InterPro (Jünemann et al., 2017; Kanehisa et al., 2022; Paysan-Lafosse et al., 2022). Amplicon sequencing is a method that quantifies strain-specific 16s ribosomal RNA (rRNA) marker genes and is a widespread method for taxonomic profiling (Jünemann et al., 2017).

Metatranscriptomics and metaproteomics give information on the transcribed genes hinting at potentially active microbial functions (Bashiardes et al., 2016; Heyer et al., 2017). Reads of transcripts are processed similarly to reads of genes in metagenomics (Bashiardes et al., 2016). In metaproteomics, the raw data consists of mass spectra of peptides, which are matched against spectral libraries or reference databases, often derived from sources like UniProt or metagenomic sequences (Heyer et al., 2017). A particular challenge in metaproteomics is mapping peptides to taxa, because different taxa may possess homologous protein domains. Therefore, peptides are either grouped, or unique peptides are considered in subsequent analyses (Schallert et al., 2022). The functional annotation of protein groups or unique peptides is then retrieved from the underlying reference database.

Metabolomics quantifies molecules below 1,500 Da, providing insights into metabolic activity (Bauermeister et al., 2021). Metabolites are identified from mass spectra using spectra libraries, while molecules can be inferred from their structural features based on NMR spectra (Liu and Locasale, 2017; Bauermeister et al., 2021). While it is feasible to quantify metabolites for the entire microbiome or its medium, linking detected metabolites to the producing species poses a challenge (Bauermeister et al., 2021). Determining metabolite pools of individual cells necessitates single-cell methods. Alternatively, chemically or isotopically labeled substrates can be added to the medium to measure the incorporation of metabolites into biomass, which indicates metabolic activity (Jehmlich et al., 2010; Hatzenpichler et al., 2020).

The primary output of metaomics methods typically comprises lists of genes or molecules alongside their respective quantities. Statistical methods aid data interpretation by revealing group differences, patterns, and correlations (Bartel et al., 2013; Yamada et al., 2020; Arıkan and Muth, 2023). Other statistical methods such as network analyses and pathway enrichment additionally provide biological contexts for metaomics data (Jiang et al., 2019; Reimand et al., 2019; Salvato et al., 2021). Data visualization facilitates comprehension of metaomics data and communication of analysis results (Gehlenborg et al., 2010; Yamada et al., 2020). Furthermore, it is possible to integrate data from two or more parallel metaomics experiments termed multiomics. Multiomics provide a holistic insight into the analyzed system rather than just one omics layer but are more expensive, and require specific experimental considerations and analysis methods (see Arıkan and Muth (2023) for a comprehensive and recent review).

The mentioned technologies allow for top-down analyses of microbiomes and their expressed and active metabolic functions. Mechanistic models with molecular resolution (Section 5) can be reconstructed, refined, validated, and integrated with metaomics data. Microbiome modeling is not limited to these data types and can exploit other omics and experimental methods depending on the utilized modeling framework. A (non-exhaustive) list of data types/methods useful for microbiome modeling and corresponding references is provided (Table 2).

**Table 2:** List of references to other (metaomics) methods that can be used in microbiome modeling.

**Data type/Method**	**References**
WGS/amplicon	Segata et al., 2013; Bragg and Tyson, 2014; Jünemann et al., 2017; Frioux et al., 2020; Zorrilla et al., 2021
metatranscriptomics	Gifford et al., 2010; Gosalbes et al., 2011; Bashiardes et al., 2016
metabolomics	Mashego et al., 2006; Zhang et al., 2012; Liu and Locasale, 2017; Bauermeister et al., 2021
enzyme activity assays	Bisswanger, 2011; Stitt and Gibon, 2014
C13-metabolic flux analysis	Wiechert, 2001; Zamboni et al., 2009; Winter and Krömer, 2013
single-cell omics	Wang and Bodovitz, 2010; Duncan et al., 2019
protein interaction data	Zhou et al., 2016
growth screenings	Oh et al., 2007; Maier and Pepper, 2015
knock out screenings and gene essentiality data	Oh et al., 2007
biomass composition	Beck et al., 2018; Lachance et al., 2019
total protein content	Noble et al., 2007; Noble and Bailey, 2009
maintenance coefficients	Stouthamer and Bettenhaussen, 1973; Vos et al., 2016
microscopy	Cesar and Huang, 2017
flow cytometry	Props et al., 2016; Hatzenpichler et al., 2020

## 5 Mathematical models are formalisms to describe biological mechanisms

Models aim to capture real-world phenomena by mathematical expressions and can be used to describe biological systems in time and space. Mathematical modeling plays a vital role in systems biology, which collects data by experimental methods, integrates, and analyzes data to obtain a holistic view of biological systems (Veenstra, 2021). Models offer significant value by integrating and compiling knowledge and complementing newly generated experimental data. They possess the capacity to make predictions, generate, and validate hypotheses. Making predictions is often cheaper than conducting experiments, and simultaneously, these predictions can inform and refine the design of experiments, making them more targeted. Additionally, modeling is essential for developing an understanding of how to control microbiomes effectively (Liu, 2023).

The explained characteristics of microbiomes (Section 3) are closely related to the questions targeted by models, such as

- What are the structures of ecological networks formed by microbiome interactions?
- Who are the important actors in these networks?
- What kinds of interactions are prevalent?
- What are the dynamics of taxonomic microbial composition and exchanged molecules?
- How do interactions influence microbiome dynamics including steady states and stability?
- What is the role of population heterogeneity and spatial organization?
- Which system inputs can be used to control the dynamics?

**Figure 2:**
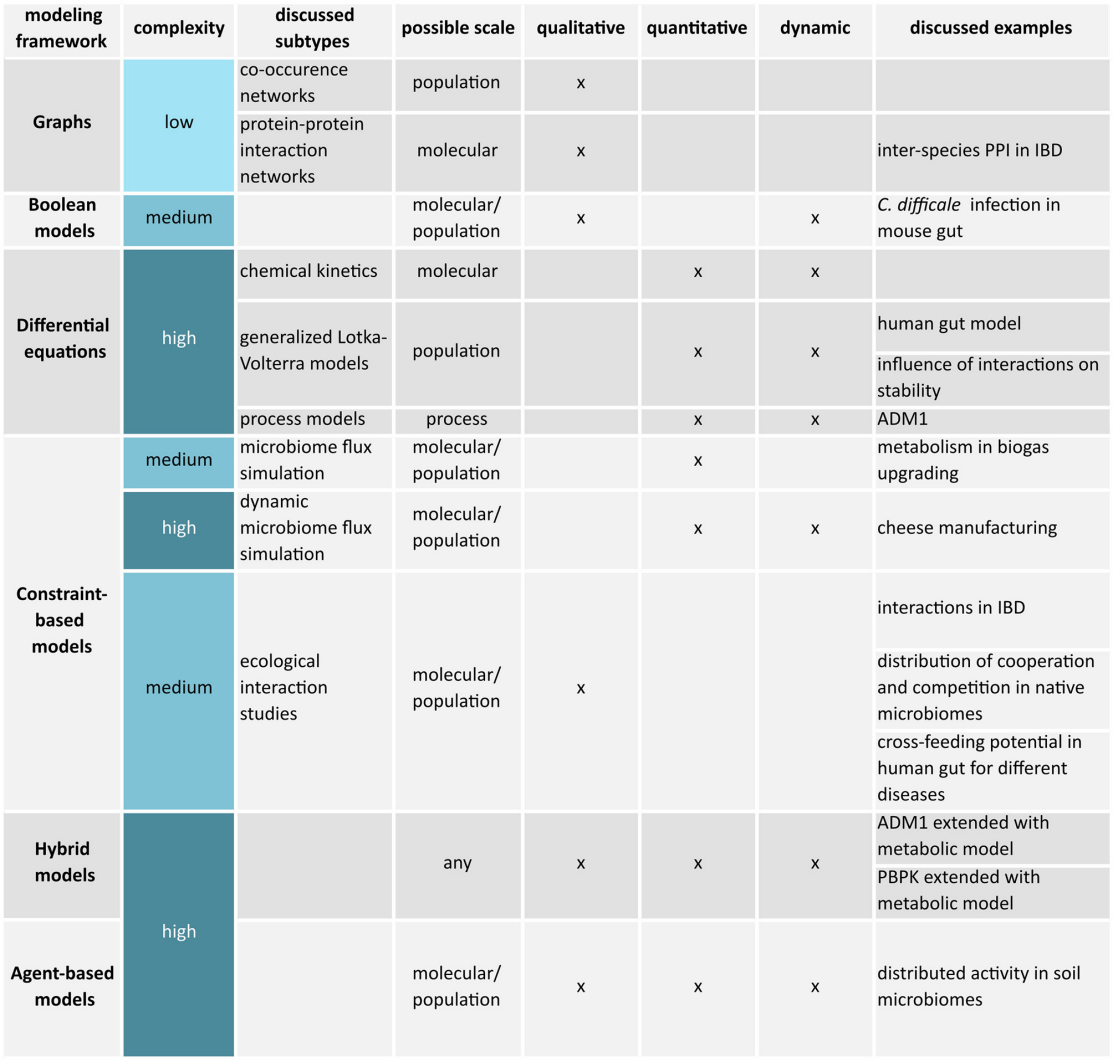
Overview of discussed microbiome modeling frameworks.

Apart from a research question, the choice of a modeling framework depends on the available data, the required mechanistic resolution, and available knowledge. This review mostly covers mechanistic models, but Section 5.1 aims to introduce the concept of statistical or machine learning-based models briefly and differentiates both paradigms. The following Sections 6 to 11 provide an overview of the most common modeling frameworks applied to understand and control microbiomes. The sections progress from simple to sophisticated frameworks also presented in the overview Figure 2. More information on modeling of biological systems and formalisms that were not considered can be found in references by Machado et al. (2011), Motta and Pappalardo (2012), and Novère (2015).

### 5.1 Statistical models and mechanistic models

Statistical models comprise a heterogeneous group of model frameworks (including machine learning models) applied to detect patterns in data, classification, or regression. These models generally capture relations between one or more input and output variables of a biological system from data (Bruggeman and Westerhoff, 2007). Assumptions on the structure (i.e., distribution, dependencies) of input and output data determine the chosen model framework (Baker et al., 2018). Adjusting model parameters to data is termed model training. The lack of mechanistic information is a disadvantage of statistical models because no information on the causal connection between input and output variables is given, models can be biased toward the structure of training data, and their range of validity is often limited (Baker et al., 2018). Statistical modeling is, for example, applied in metaproteomics to improve protein identification (Bouwmeester et al., 2020), predict disease states from metagenomes (Pasolli et al., 2016), or for the detection of potential disease biomarkers (Tang et al., 2020) and biomarker panels (Sydor et al., 2022). A simple example of statistical modeling is fitting a calibration curve to data from a colorimetric protein assay by linear regression (Ninfa et al., 2009). Reviews by Pasolli et al. (2016) and Hernández Medina et al. (2022) are recommended to obtain an overview on the application of statistical models to microbiomes.

Contrary to statistical models, mechanistic models can represent physiological processes in (more or less resolved) detail (Baker et al., 2018). Mechanistic modeling typically requires less data than statistical models but demands a thorough understanding of the components of a biological system. The great advantage of mechanistic models is their display of causality. Additionally, model entities and model parameters can be integrated with (meta)omics measurements. However, mechanistic models rely on simplifying assumptions (e.g., metabolic steady state or the homogeneity of cell populations), limiting their applicability. Moreover, the process of building models can be laborious, involving iterative cycles of validating model predictions against experimental data and model refinement (Novère, 2015).

## 6 Graphs can represent ecological and molecular interactions

Biological systems consist of interacting parts and thus inherit a network structure. Such networks can be represented mathematically by graphs that embed biological entities or environmental factors (e.g., molecules, species, pH, temperature) as nodes and their interactions as edges (Layeghifard et al., 2017; Koutrouli et al., 2020). Edges can be undirected to represent associations (e.g., molecule A binds with molecule B, species A occurs with increased pH value) or directed to indicate a flow of mass (e.g., metabolite A is catalyzed by reaction Y to metabolite B), (in)activation (e.g., protein A activates/inhibits protein B), or whether interactions are dynamic (e.g., species A grows with a delayed response to the increase in pH) (Layeghifard et al., 2017; Koutrouli et al., 2020). Graphs are qualitative models because they only explain relationships between biological entities.

Graphs can be expressed as adjacency matrices containing a row and column for each node, with matrix entries representing the occurrence and the type of an interaction (Samaga and Klamt, 2013; Koutrouli et al., 2020). The analysis of graphs provides information on the organization of biological networks, for example, whether the network has a modular organization (Koutrouli et al., 2020). Metrics such as node degree (number of edges connected to a node) and betweenness centrality (number of paths going through a node/edge) can respectively highlight molecular hubs or potential metabolic bottlenecks (Koutrouli et al., 2020). Furthermore, for networks representing signal flow, paths (routes between input and output) and feed-forward or feed-back loops can be uncovered to obtain hints on the dynamic behaviors of networks (Samaga and Klamt, 2013; Koutrouli et al., 2020).

Subsequently, co-occurrence networks and inter-species protein-protein interaction networks are given as application examples for graph analysis of microbiomes. However, methods for graph analysis can be applied to any model that incorporates a network structure (e.g., generalized Lotka-Volterra models, Section 8.1 or genome-scale metabolic reconstructions, Section 9). The flexible structure of graphs also allows for storage and analysis of data in graph databases and knowledge graphs (Santos et al., 2022; Walke et al., 2023).

Recently, a comprehensive review on the application of graphs to microbiomes has been published (Liu et al., 2020). Readers interested in graph theory applied to biological networks in general are referred to reviews by Pavlopoulos et al. (2011) and Koutrouli et al. (2020). Multiple software packages are available for general purpose or biological graph analysis (Table 3 and review by Liu et al., 2020).

**Table 3:** List of graph analysis software. Many other tools are listed in the review by Liu et al. (2020).

**Software (implementation)**	**Application**	**References**
NetworkX (Python)	General-purpose graph analysis	Hagberg et al., 2008
Gephi (desktop application)	General-purpose graph analysis	Bastian et al., 2009
GraphViz (command line)	Graph layout calculation	Ellson et al., 2002
Cytoscape (desktop application)	General-purpose graph analysis	Shannon et al., 2003
CoNet (Cytoscape plugin)	Reconstruction of co-occurence networks	Faust and Raes, 2016
iNAP (web application)	Reconstruction and analysis of co-occurence networks	Feng et al., 2022
MicrobioLink (Python)	Inter-species pathway inference from PPI and signaling networks	Andrighetti et al., 2020
TieDIE (Python)	Pathway inference from PPI and signaling networks	Paull et al., 2013

### 6.1 Co-occurence networks

Co-occurrence networks are coarse-grained representations of species (or operational taxonomic units, OTUs) as nodes, and their associations as undirected edges (Layeghifard et al., 2017; Liu, 2023). These networks can be reconstructed from microbiome composition data (e.g., tables of 16s rDNA gene counts across multiple samples) (Faust and Raes, 2016). After preprocessing steps, such as normalization to total counts in a sample (Layeghifard et al., 2017), the input data undergo inference algorithms to predict associations between species. Simple inference algorithms use correlation (e.g., Pearson or Spearman correlation) to infer associations between species (Layeghifard et al., 2017). Consequently, network edges represent pairwise correlations between two nodes. Weak associations in the network can be filtered out by setting a threshold for the used association metric (Faust and Raes, 2016). Additionally, environmental factors such as pH, temperature, and oxygen concentration can be included as individual nodes in the network (Faust and Raes, 2016). The accuracy of predicted interactions depends on the chosen inference algorithm, as shown by Hirano and Takemoto (2019).

Edges of the resulting network can be the sum of several ecological interactions (e.g., cross-feeding, antimicrobial peptides, etc.) or higher order interactions [i.e., interactions of more than two species (Ludington, 2022)] making it difficult to infer the exact interaction mechanism. Furthermore, edges can be caused by indirect associations, for example, due to preference for the same ecological niche (Heyer et al., 2016), emphasizing the principle that “correlation is not causation” (Hirano and Takemoto, 2019; Liu, 2023). Despite these limitations, graph theoretical analysis of co-occurrence networks can provide insight into microbiomes as reviewed by Layeghifard et al. (2017) and Kumar et al. (2019). For instance, cluster analysis can identify co-associated species by finding densely connected nodes within their cluster but with fewer links to nodes outside their cluster (Layeghifard et al., 2017). The importance of individual species nodes can be predicted from their centralities (e.g., degree, or betweenness centrality), node influence, or link analysis (Layeghifard et al., 2017).

Reviews by Layeghifard et al. (2017), Röttjers and Faust (2018), and Kumar et al. (2019) are recommended for in-depth information on the inference and analysis of co-occurrence networks.

### 6.2 Inter-species protein-protein interaction networks

Edges in co-occurrence networks may represent convoluted molecular interactions, such as metabolic interactions (covered in Section 9), and inter-species protein-protein interactions (PPIs), exemplified in this section. Other molecular network types, like regulatory networks, are not explicitly addressed because, to our knowledge, network analysis has not been applied to these network types within the context of microbiomes. Other (molecular) network types and their analysis are reviewed by Winterbach et al. (2013) and Koutrouli et al. (2020). An interactive introduction to graph theory for PPIs is available at (https://doi.org/10.6019/tol.networks_t.2016.00001.1).

Microbiome-derived proteins can modulate host signaling and are implicated in health and diseases such as inflammatory bowel disease (IBD) and colorectal cancer (CRC) (Fischbach and Segre, 2016; Andrighetti et al., 2020; Zhou et al., 2022). Information on interacting proteins is obtained experimentally (Zhou et al., 2016) and can be predicted from sequence or structural similarity, or molecular simulations (Skrabanek et al., 2007; Zhou et al., 2022). Public databases like String (Szklarczyk et al., 2020) or IntAct (del Toro et al., 2021) offer access to protein interactions or molecular interactions, respectively. PPI data are used to reconstruct signaling networks, archived in databases such as OmniPath (Türei et al., 2016), Reactome (Gillespie et al., 2021), or WikiPathways (Martens et al., 2020).

Andrighetti et al. (2020) leveraged inter-species PPI networks to identify potential signaling pathways in hosts modulated by microbiome-derived proteins. Their MicrobioLink pipeline allows users to input metaproteins and target proteins or genes in hosts putatively influenced by the microbiome. Predicted microbiome-host protein interactions (source set) and putative targets (target set) are subjected to the TieDIE method, which utilizes network diffusion (Paull et al., 2013). The additional input of network diffusion is a directed network (e.g., a signaling network), containing relevant and non-relevant pathways. The network diffusion algorithm propagates a relevance score across the network from the source and target sets, expanding them to include new nodes. Nodes present in both sets are potential contributors to pathways of interest, which can be further filtered to extract condition-specific pathways (Paull et al., 2013; Andrighetti et al., 2020). Using MicrobioLink, Andrighetti et al. (2020) identified metaproteins potentially interacting with pathways regulating autophagy in Crohn's disease (CD), a form of IBD.

### 6.3 Benefits and limitations of graph models

The examples above illustrate the versatility of graphs in representing systems with interacting components. Graphs can incorporate multiple node and edge types, and even weighted edges (Koutrouli et al., 2020). They can be constructed from experimental data or inferred from abundance data obtained through (meta)omics methods. Despite requiring relatively little information, graphs can reveal insightful properties of biological systems. While many graph methods are limited to data interpretation, some, like link prediction and perturbation analysis, can forecast future behaviors or system properties (Koutrouli et al., 2020). Link prediction anticipates future edges or missing links, and could potentially predict emerging interactions in ecological networks. Perturbation analysis assesses the impact of disturbances on network behavior, offering insights into the effects of species removal in ecological networks (Koutrouli et al., 2020). Moreover, as graphs are universally applicable, algorithms developed for other domains, like social networks, could be leveraged for biological graphs.

However, graphs have limitations. They are qualitative and cannot predict molecular or species abundances. Additionally, as graphs are static, they cannot simulate the time evolution of dynamic systems. Nevertheless, time-series data could be analyzed by creating separate networks for each time-point and investigating changes in network properties over time.

## 7 Boolean modeling in microbial ecology

Edges in signaling networks typically represent the activation or inhibition of molecules. Similarly, edges in ecological networks can represent the inhibition or promotion of one species by another. A corresponding expression could be: “Species A is present **if** species B is present.” Such expressions are compiled in Boolean models, commonly applied to cellular signaling and gene regulation (Wang et al., 2012; Barbuti et al., 2020) but have also been used in one instance for microbiome modeling (Steinway et al., 2015). Boolean models are based on variables with binary activation states (e.g., zero or one) corresponding to genes, signaling molecules, or the presence of species in a microbiome. Activation states are updated by Boolean expressions linking all activating/inhibiting interactions from other variables, enabling dynamic simulations of biological systems (Wang et al., 2012; Barbuti et al., 2020).

Boolean models are qualitative because they represent relations of state variables and activation states while omitting molecular quantities. They are useful when kinetic parameters for models based on differential equations are difficult to determine (Section 8) (Machado et al., 2011). Typical analyses of Boolean models explore dynamic behaviors or steady states (Samaga and Klamt, 2013). Dynamic simulations require a time-scale separation of fast and slow processes due to the discrete updating scheme of state variables. For further information on Boolean models, see articles by Karlebach and Shamir (2008), Wang et al. (2012), Samaga and Klamt (2013), or Barbuti et al. (2020). Software for Boolean modeling is listed in Table 4.

**Table 4:** List of software for Boolean modeling.

**Software (implementation)**	**Application**	**Reference**
BoolNet (R)	Reconstruction from time-series data and simulation of Boolean networks	Müssel et al., 2010
BooleanNet (Python)	Simulation of Boolean networks	Albert et al., 2008
PyBoolNet (Python)	Simulation of Boolean networks	Klarner et al., 2016
The Cell Collective (web application)	Exchange and simulation of Boolean networks	Helikar et al., 2012
CoLoMoTo Interactive Notebook (Python)	Containerized collection of Boolean modeling software	Naldi et al., 2018

To our knowledge, Steinway et al. (2015) are the only researchers who developed a Boolean model for microbial ecology to date. They explored the population dynamics of a mouse gut microbiome infected with *Clostridium difficile* after antibiotic treatment as well as therapeutic interventions. Using 16s rRNA gene abundance time-series data from a mouse study, a Boolean network was inferred, where each node represents a genus, its state indicating presence (one) or absence (zero), and edges representing inhibitory or promoting relationships. Additionally, an abiotic node representing the presence or absence of the antibiotic was introduced.

Attractor analysis was employed to explore the steady states of the system. To this end, a vector of initial state variables is defined, and then the model is updated until all state variables stabilize (i.e., a steady state is reached) or oscillate. This is repeated for all possible initial states to identify attractors, i.e., steady states attracting a given set of initial conditions. Attractors are interesting because they correspond to known phenotypes of a biological system (Barbuti et al., 2020). Steinway et al. (2015) identified 21 attractors, including six consistent with experimentally inferred microbiome compositions, i.e., the healthy microbiome, the microbiome after treatment, and the infected microbiome after treatment.

To identify potential treatments for *C. difficile* infection, perturbation analysis was conducted. Initially, the steady states of attractors representing the microbiome after antibiotic treatment and the *C. difficile* infected microbiome after treatment were used as new initial states. Subsequently, an evaluation was performed to determine which state variables needed to be activated or knocked out to restore the healthy state. From this analysis, *Lachnospiraceae* and *Barnesiella* were identified as candidates needing activation to inhibit *C. difficile*, corresponding to probiotic treatment with these genera (Steinway et al., 2015).

Furthermore, the authors created genome-scale reconstructions of metabolism (Section 9) for representative species to investigate whether metabolic interactions contribute to inhibition or promotion of *C. difficile* growth. These reconstructions enabled the identification of metabolic “inputs” and “outputs” used to evaluate scores for pairwise competition or mutualism. *C. difficile* and *Barnesiella* exhibited low competition and high mutualism scores, indicating non-metabolic mechanisms for the inhibition of *C. difficile* by *Barnesiella*, a finding supported by co-culture experiments.

### 7.1 Benefits and limitations of Boolean models

Being qualitative but capable of dynamic simulations is a benefit and limitation of Boolean models. The ecological model presented enables dynamic analyses without necessitating many parameters, which can be challenging to infer. In contrast, quantitative dynamic models like the generalized Lotka-Volterra model rely on such parameters, which can be difficult to extract from data (Section 8.1). Additionally, Boolean models can be constructed with minimal qualitative data, and their analysis is computationally less complex compared to differential equation-based models (Barbuti et al., 2020). These characteristics were also key factors in the decision of Steinway et al. (2015) to adopt this framework. Hence, Boolean models are viable for reconstructing larger ecological networks of microbiomes. They can also serve as starting points for dynamic modeling, as their predictions often align with those of differential equation models and can be extended to such quantitative models (Albert and Thakar, 2014). Moreover, they could become the preferred framework for simulating genome-scale networks of signaling and regulation (Romers et al., 2020), or hybrid models that integrate metabolism, signaling, and regulation (Section 10).

The qualitative nature of Boolean models poses several challenges. Continuous time-series data used for modeling have to be discretized, for example, through thresholding or clustering methods (Albert and Thakar, 2014; Steinway et al., 2015). Molecular processes, such as in signaling and regulation, may span several time scales, which requires a separation of fast and slow processes or specific updating schemes (Saez-Rodriguez et al., 2007; Albert and Thakar, 2014; Münzner et al., 2019).

## 8 Differential equations—Quantitative and dynamic models of biological systems

Differential equations can model dynamic systems at any scale and complexity. Ordinary differential equations (ODEs) express quantitative changes in biological entities (e.g., metabolites, biomass) over time. Spatially resolved models require partial differential equations (PDEs).

**Figure 3:**
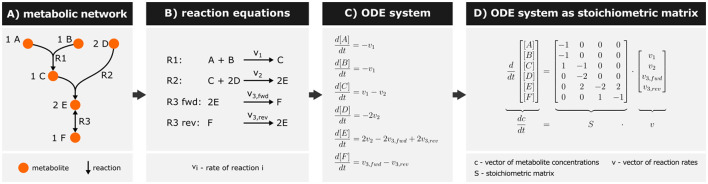
Example for representing a metabolic network mathematically. **(A)** Representation of a metabolic network as a hypergraph (i.e., edges can connect more than two nodes). The stoichiometric coefficients are denoted in front of the metabolite name. Reversible reactions are indicated by a double-headed arrow (R3). **(B)** The network is represented through reaction equations, with the reversible reaction R3 separated into forward and reverse reactions. **(C)** An Ordinary Differential Equation (ODE) system is formulated describing mass balances for each metabolite. Square brackets denote metabolite concentrations. **(D)** The ODE system is then represented as a stoichiometric matrix *S*, where rows correspond to metabolites and columns to reactions. Matrix entries reflect the stoichiometries of metabolites involved in respective reactions.

The kinetics of a metabolic network is a prime example to explain the structure of ODE models (Figure 3). Metabolic networks consist of enzyme-catalyzed biochemical reactions that transform (and transport) metabolites (Figure 3A). Each reaction operates at a rate *v*_*i*_ defining the molecular turnover of that reaction (Figure 3B). ODEs describe changes in metabolite concentrations by these reactions and represent mass balances. Thereby, model equations include terms for the reaction rates of metabolite production and consumption, multiplied by their respective stoichiometric coefficients (Mendes et al., 2009) (Figure 3C).

The resulting ODE system can be written as a matrix expression (Figure 3D), wherein the stoichiometric matrix *S* decodes the network topology. In this matrix, metabolites are represented as rows, reactions as columns, and the stoichiometric coefficients of a metabolite in each reaction as entries. Multiplying the stoichiometric matrix by the vector of reaction rates *v* yields the original system of ODEs (Novère, 2015; Gottstein et al., 2016).

The rate of enzymatic reactions, *v*, depends on factors like temperature, pH, and metabolite and enzyme concentrations. However, metabolic models typically simplify this dependency by utilizing the Michaelis-Menten equation. This equation accounts only for the influence of substrate and enzyme concentration on reaction rate and models how enzyme saturation increases with rising substrate concentration, shown for the example reaction R3 fwd (Equation 1, *v*_*max*_ - maximal reaction rate of forward reaction R3, *K*_*m*_ - Michaelis constant, [*E*] - concentration of substrate E, *k*_*cat*_ - enzyme turnover number, *e*- enzyme concentration) (Chen et al., 2010).


(1) $$\begin{array}{l} v_{3,fwd}=\frac{v_{max}}{K_{M}+\left[E\right]}\left[E\right]=\frac{k_{cat}\cdot e}{K_{M}+\left[E\right]}\left[E\right] \end{array}$$

Metabolite concentrations (Figures 3C, D) are continuous variables describing the system's state (i.e., state variables), whereas *v*_*max*_ and *K*_*M*_ denote system-specific kinetic parameters that can be retrieved from databases such as BRENDA or Sabio-RK (Wittig et al., 2017; Chang et al., 2020). Additionally, the experimenter can define parameters related to the experimental setup, such as the dilution rate in continuous bioreactor cultivation (see Garza et al., 2023 for an example model). In many cases, parameter values are not readily available in databases. In that case, dedicated experiments such as enzyme assays can be performed to obtain biological parameters such as *v*_*max*_ and *K*_*M*_ values (Bisswanger, 2011). Alternatively, the model itself can be used to estimate parameters directly from available experimental data.

The input for parameter estimation is experimental data [e.g., time-series or steady state data (Ashyraliyev et al., 2009; Villaverde et al., 2018)] and a model with an initial set of (random) parameters and initial values for state variables. The model is then used to predict the experimental data, and the disagreement between prediction and data is quantified (Ashyraliyev et al., 2009; Mendes et al., 2009; Villaverde et al., 2018). Optimization methods are then employed to adjust parameter values (and initial values) to minimize these discrepancies. For large and non-linear model equations, multiple sets of parameters may exist that achieve minimal disagreement (i.e., parameter sets fulfilling local optima exist) (Ashyraliyev et al., 2009; Villaverde et al., 2018). The linear least squares algorithm employed in linear regression is an example of parameter estimation. Parameter estimation, also known as parameter fitting or model training, is similar to techniques used in statistical modeling. The uncertainty of parameters can be assessed by statistical methods, which are reviewed by Marino et al. (2008).

The analysis of ODE systems originates from systems theory. Common methods relevant to microbiome analysis include time-course simulation, steady state analysis, bifurcation, and sensitivity analysis. Similar to Boolean models, time-course simulations necessitate initial values for state variables (e.g., initial metabolite concentrations) and a time horizon. Instead of Boolean rules, the evolution of state variables is calculated by numerical integration of the ODE system over the time horizon (Mendes et al., 2009). In short, numerical integration is an iterative process that divides the time horizon into small time steps. Integration algorithms such as the Runge-Kutta method start at the beginning of the time horizon and utilize state variables in the current step to estimate their values in the next step using the differential equation system (Butcher, 2000). Alternatively, probabilistic algorithms like the Gillespie algorithm can address stochastic events for simulating few molecules (Mendes et al., 2009).

Steady state analysis involves determining stable or unstable steady states (Mendes et al., 2009; Layek, 2015). A dynamic system is in a steady state when its state variables remain constant over time, i.e., the differentials in the ODE system become zero, yielding Equation (2) for the metabolic network.


(2) $$\begin{array}{l} \frac{dc}{dt}=0=S\cdot v \end{array}$$

Multiple steady states can exist, meaning there could be several values for *v* satisfying the Equation (2). Software tools like Copasi (Mendes et al., 2009) numerically determine these values. Equation (2) is also the core of flux balance analysis, a method from constraint-based modeling, which will be explained in Section 9.

Bifurcation analysis examines how steady states (and trajectories) change with variations in system parameters and identifies the parameter values where these changes occur (Layek, 2015). This analysis is interesting for optimizing biological processes such as biogas production or describing signaling and regulatory network “switching” between states (Aldridge et al., 2006b; Bornhöft et al., 2013).

Sensitivity analysis assesses the system's susceptibility to parameter values and initial conditions (Aldridge et al., 2006a; Mendes et al., 2009). It can be performed by varying individual parameter values and quantifying the relative change of a model output or objective function (Aldridge et al., 2006a; Zi, 2011). Sensitivity analysis helps determine required parameter accuracies, identifies relevant parameters for achieving objectives (e.g., product maximization), and evaluates the biological system's robustness (Mendes et al., 2009). Parameter scanning is a similar procedure in which the model output is determined over a range of parameter values (Mendes et al., 2009).

Next, dynamic model examples relevant to microbiomes are presented, including dynamic population models and process models. Dynamic analyses based on constraint-based models such as dynamic flux balance analysis are discussed in Section 9.2.2. For further reading on dynamical systems theory, readers can explore books by Layek (2015) and Hirsch et al. (2012). Software for dynamic modeling is listed in Table 5.

**Table 5:** List of software for dynamic modeling.

**Software (implementation)**	**Application**	**References**
Matlab	Programming language with integrated numerical methods	The MathWorks Inc., 2024
Tellurium (Python)	Dynamical modeling of biological systems	Choi et al., 2018
Copasi (desktop application)	Dynamical modeling of biological systems	Mendes et al., 2009
PySCeS (Python)	Dynamical modeling of biological systems	Olivier et al., 2004
MDSINE (Matlab)	Inference of gLV interaction parameters from time-series data	Bucci et al., 2016
BEEM-static (Python)	Inference of gLV interaction parameters from steady-state data	Li et al., 2021
miaSim (R)	Dynamic modeling of microbiomes	Gao et al., 2023
Web-gLV (web application)	Inference of gLV interaction parameters from time-series data and simulation	Kuntal et al., 2019

### 8.1 Population models based on differential equations

ODE-based population models of microbiomes focus on the dynamics of species abundances. In the review by Liu (2023), dynamic population models were categorized into species-only models and mediator-explicit models. Species-only models account for direct interactions among species but do not consider the mode of action (e.g., interactions via metabolites or signaling molecules). Thus, species-only models share a similar level of mechanistic detail with co-association networks and the previously discussed Boolean population model.

The biggest drawback of species-only models is their limitation to pairwise interactions with linear effect on species abundances, the lack of information on interaction mechanisms, and no incorporation of host organisms (Liu, 2023). Furthermore, they are effective, meaning that they are specific to the dataset they were built on Liu (2023). Mediator-explicit models, such as consumer-resource models, not only incorporate species abundances but also consider the concentration of mediator molecules (e.g., metabolites and signaling molecules) and their impact on growth. These models provide a deeper mechanistic resolution but are challenging to parameterize and therefore difficult to apply in practice (Liu, 2023).

The generalized Lotka-Volterra (gLV) model is species-only and among the most popular model types for microbiomes (Gonze et al., 2018). It accounts for changes in species abundance by balancing growth and pairwise stimulative or inhibitory interactions (Gonze et al., 2018; Liu, 2023) (Equation 3, adapted from Liu, 2023, [*X*_*i*_], [*X*_*j*_] - species abundances, *r*_*i*_ - intrinsic growth rate, *a*_*ij*_ - pairwise interaction factor).


(3) $$\begin{array}{l} \frac{d\left[X_{i}\right]}{dt}=\left[X_{i}\right]\left(r_{i}+\overset{N}{\underset{j=1}{\sum }}a_{ij}\left[X_{j}\right]\right) \end{array}$$

The parameters of gLV models have been determined in a bottom-up manner from laboratory experiments for communities of up to 12 species (Venturelli et al., 2018; Liu, 2023). Alternative data-driven approaches are suited for larger microbiomes and can infer parameters from time-series or steady state abundance data from different formulation: microbiomes (i.e., 16s rRNA gene counts) (Bucci et al., 2016; Xiao et al., 2017; Liu, 2023). Liu (2023) extensively discusses the advantages and caveats of both data types as well as algorithms for parameter inference.

Venturelli et al. (2018) applied gLV models to explore prevalent interaction types in microbiomes and their influence on human gut microbiome assembly. They conducted mono-, pairwise, and multi-species cultivation experiments to determine gLV parameters for a synthetic microbiome comprising 12 representative species. Utilizing a least squares algorithm, they fitted their model to training sets of time-series data. By training their model on different datasets, such as mono-culture only or mono-culture and pairwise culture, they assessed the informational content of the datasets. Parameters trained on pairwise data effectively explained data from the full 12-species microbiome, suggesting that pairwise interactions govern most microbiome interactions. Utilizing the trained interaction factors *a*_*ij*_, the authors reconstructed the ecological network, revealing mostly negative and few positive microbial interactions. The authors identified species with similar interaction patterns, important hub species, and species whose fitness depended on the microbiome. Additionally, they investigated the dependence of microbiome composition on initial species compositions (i.e., history-dependence). To this end, they performed time-course simulations for interacting species, varying initial biomass abundances and interaction strengths. They discovered that history dependence for pairwise negative interactions frequently arises due to slow system relaxation into a steady state.

Other studies, such as that by Coyte et al. (2015), investigate the effect of interactions on microbiome properties, such as stability. Coyte et al. (2015) developed a framework based on the gLV model, enabling them to sample interaction parameters for any number of species and connectivity. They assessed the stability of an arbitrary steady state of the model microbiome using a systems-theoretic approach, utilizing Eigenvalues of the system's Jacobian matrix (see supplementary of Coyte et al. (2015) or Layek (2015), p. 194, for a simpler example). The authors found that many cooperative interactions destabilize microbiomes due to the coupling of species growth, while competitive interactions could introduce stability by dampening this effect. This was also in line with results from Venturelli et al. (2018). It was also found, that increased species diversity generally decreases stability but can be counteracted by competitive interactions.

For further insights into ecological modeling, interested readers are directed to reviews by Gonze et al. (2018), van den Berg et al. (2022), and Liu (2023).

### 8.2 Dynamic process models

While population models focus on the molecular-scale interactions within microbiomes, process models examine the effects of microbiomes on the scale of production systems or ecosystems (Muñoz Tamayo et al., 2010; Hauduc et al., 2013; Sulman et al., 2014; Wieder et al., 2015; Santos et al., 2020). These models are manually constructed and intended to be used in process design, optimization, and control (Batstone et al., 2002), resulting in reduced mechanistic resolution. Process models have been utilized to simulate carbohydrate degradation in the human colon (Muñoz Tamayo et al., 2010), nutrient removal from wastewater by activated sludge (ASM model) (Hauduc et al., 2013; Santos et al., 2020), and model nutrient cycling in the environment (Wieder et al., 2015).

Process modeling and analysis are explained using the anaerobic digestion model 1 (ADM1) as an example. ADM1 is a macroscopic process model developed specifically for the anaerobic production of biogas. It describes the step-wise degradation of complex organic matter to biogas (*CO*_2_ and methane) by microbial processes using differential and algebraic equations (Batstone et al., 2002). The model incorporates biochemical reactions for the degradation of organic matter and physicochemical processes (e.g., ion association/dissociation and gas-liquid transfer). Seven biochemical reactions modeling the degradation of key compounds are linked to the accumulation and death of microbial biomass. State variables of the model describe the concentration of resolved and gaseous chemical compounds (e.g., monosaccharides and methane gas) and biomass of functional microbial groups (e.g., sugar and amino acid degraders) (Copp et al., 2003).

ADM1 was originally intended for application in biogas plant design and operation, process optimization, and control, as well as serving as a foundation for further model development (Batstone et al., 2002). For example, Ozgun (2019) trained ADM1 to data from a biogas plant using municipal wastewater sludge, aiming for future process optimization. Additionally, Waszkielis et al. (2022) extended ADM1 to a biogas process utilizing maize silage and manure as substrates, identifying influential parameters for the process and variables for process monitoring. Further applications are discussed by Batstone et al. (2006).

Several simulation studies have been conducted using ADM1. Bornhöft et al. (2013) performed simulation studies to investigate process stability through bifurcation analysis. They identified steady states corresponding to desired process operation and explored the influence of varying parameters such as substrate inlet concentrations and dilution rates. Parameter ranges were determined where the system could maintain its steady state, predicting regions suitable for safe plant operation. Additionally, using ADM1, they could elucidate mechanisms destabilizing the process beyond safe parameter regions.

Dynamic models are also employed to guide process control, such as in model predictive control (Section 11). The original ADM1 is deemed to be impractical for this purpose due to its complexity, necessitating simpler and more robust models for simulations with fewer parameters to calibrate (Weinrich and Nelles, 2021; Weinrich et al., 2021). In a recent study, Weinrich and Nelles (2021) developed a model simplification strategy, which combines multiple degradation reactions from the original ADM1 into simplified reaction equations. This resulted in four models of varying complexity, which were validated in a parallel study using data from lab biogas reactors, showing similar accuracy to the original ADM1 (Weinrich et al., 2021).

Another way to reduce computational demand is to learn the behavior of mechanistic models with machine-learning-based surrogate models (Gherman et al., 2023). A surrogate model is a black box model which only considers inputs and outputs of a biological system, while omitting mechanistic information. Wagner and Schlüter (2020), for example, applied a deep neural network to learn the ADM1. To this end, they trained the neural network on simulation data of the original model and could predict steady states and methane production time courses with accuracies above 96%. The resulting surrogate model was then used with model predictive control to control methane production. Due to the flexibility of machine learning, surrogate modeling could also be applied to other mechanistic model types.

### 8.3 Benefits and limitations of dynamic models

Differential equations offer high flexibility and can be applied to model dynamical systems of varying scale, with the capacity to resolve models spatially. They can constitute simple but powerful models such as the gLV model but can be extended to arbitrary complexity. State variables are continuous but can be simulated stochastically by the Gillespie algorithm. Even if parameters are unavailable, ODE models can be used to sample the parameter space and investigate general system properties (Coyte et al., 2015; Liu, 2023). Systems theory provides comprehensive analysis methods characterizing system dynamics. Furthermore, dynamic models are not limited to predictive studies but can be used for process design, optimization, and control as exemplified by the ADM1 model.

Differential equations are among the most complex model types. Building and analyzing such models demands knowledge of system theory and may not be as intuitive for beginners compared to other frameworks. However, scientific communities established standard models such as gLV models or ADM1. Differential equations depend on the availability and accuracy of parameters. While parameters can be fitted to experimental data, it can be challenging to determine the required information content and amount of data (Liu, 2023). Models with many parameters (over-parametrization), as well as scarce and erroneous data, are further challenges for parameter estimation (Gábor and Banga, 2015). Moreover, optimization algorithms for parameter estimation may not find the most optimal parameter set (Gábor and Banga, 2015). Lastly, the analysis and simulation of differential equations depend on numerical methods that can run into instabilities and are computationally expensive (Butcher, 2000).

## 9 Constraint-based modeling of microbiomes

Metabolic networks can be reconstructed from the annotated genome of an organism (Section 9.1) resulting in genome-scale metabolic reconstructions containing thousands of metabolic reactions (Heinken et al., 2023). Theoretically, such networks could be transferred into dynamic models as described (Figure 3). However, the availability and accuracy of kinetic parameters such as *k*_*cat*_ or *K*_*m*_ are limited. For instance, the BRENDA database contains approximately 180,000 *K*_*m*_ values (date of access April 9, 2024, https://www.brenda-enzymes.org/statistics.php) while NCBI lists over 700,000 sequenced prokaryotic genomes (date of access April 9, 2024, https://www.ncbi.nlm.nih.gov/genome/browse#!/prokaryotes/), each potentially containing a few thousand enzymatic reactions per organism. Determining these parameters involves laborious enzyme assays performed on isolated enzymes, which can be challenging to obtain for species found only within microbiomes (Wright et al., 1992; Bisswanger, 2011; Thornbury et al., 2019). Moreover, enzyme parameters may deviate from *in vivo* values (Wright et al., 1992) or are often not reached *in vivo* (Bekiaris and Klamt, 2020). Furthermore, it can be challenging to identify parameters unambiguously from available data (Berthoumieux et al., 2012). Constraint-based modeling offers a solution to these challenges by omitting kinetic parameters. The subsequent sections explain the reconstruction process of genome-scale metabolic reconstructions for microbiome members (Section 9.1) as well as constraint-based modeling of microbiomes (Section 9.2).

Genome-scale reconstructions and constraint-based models are widely utilized in microbial ecology, (environmental) biotechnology, and life sciences. They are used to investigate ecological interactions in microbiomes (Machado et al., 2021; van Leeuwen et al., 2023), optimize the production of chemicals, design stable synthetic microbiomes (García-Jiménez et al., 2021), investigate the degradation of pollutants (Xu et al., 2018), design microbiomes for optimal immune system modulation (Stein et al., 2018), and drug discovery (Curran et al., 2020). Constraint-based modeling and its applications have been discussed in many comprehensive reviews and only a few examples are covered here. Reviews by Biggs et al. (2015) and Heinken et al. (2021) are recommended for overviews and history of constraint-based microbiome modeling, Kumar et al. (2019) and Garza et al. (2023) focus on modeling of the human gut, García-Jiménez et al. (2021) provide a deep overview focused on biotechnological and engineering methods, and Gottstein et al. (2016) provide a great theoretical background. Scott et al. (2023) provide an overview and benchmarking of software utilizing genome-scale reconstructions. A list of software for creating genome-scale reconstructions, and qualitative and quantitative analyses is provided in Table 6.

**Table 6:** List of software for genome-scale metabolic reconstruction and constraint-based modeling.

**Software (implementation)**	**Application**	**References**
COBRA (Matlab)	Genome-scale reconstruction and constraint-based modeling	Heirendt et al., 2019
RAVEN (Matlab)	Genome-scale reconstruction and constraint-based modeling	Wang et al., 2018
CarveME (Python)	Genome-scale reconstruction	Machado et al., 2018
gapseq (R)	Genome-scale reconstruction	Zimmermann et al., 2021
CarveME (Python)	Genome-scale reconstruction	Machado et al., 2018
KBase (web application)	Genome-scale reconstruction and constraint-based modeling	Machado et al., 2018
COBRApy (Python)	Constraint-based modeling	Ebrahim et al., 2013
CellNetAnalyzer (Matlab)	Constraint-based modeling	von Kamp et al., 2017
SteadyCom (Matlab)	Microbiome FBA	Chan et al., 2017
MICOM (Python)	Microbiome FBA	Diener et al., 2020
μBialSim (Matlab)	Microbiome dFBA	Popp and Centler, 2020
SMETANA (command line)	Potential for cooperative and competitive interactions	Zelezniak et al., 2015
GECKO (Matlab)	Enzyme-constrained modeling	Domenzain et al., 2022
AutoPACMEN (Python, Matlab)	Enzyme-constrained modeling	Bekiaris and Klamt, 2020
tINIT/ftINIT (Matlab)	Contextualization of genome-scale reconstructions	Gustafsson et al., 2023, Agren et al., 2014
MEMOTE (command line)	Quality testing of constraint-based models	Lieven et al., 2020
CobraMod (Python)	Curation of constraint-based models	Camborda et al., 2022

### 9.1 Reconstructing microbiome metabolism

Genome-scale metabolic reconstructions provide detailed resolution of metabolism at the level of individual metabolites and enzymatic reactions. The reconstruction process uses an annotated whole genome sequence of one organism as input and typically follows the procedure proposed by Thiele and Palsson (2010). The first step is usually automated and retrieves for each gene reactions and associated metabolites from biochemical or dedicated databases for modeling such as KEGG (Kanehisa et al., 2022), ModelSEED (Seaver et al., 2020), or BiGG (King et al., 2015). The resulting draft reconstruction contains lists of metabolic genes, reactions, and metabolites and is manually curated and converted into a constraint-based model (Section 9.2). Such models can be used to predict growth phenotypes, substrate utilization, production of metabolites, and growth rates, which can be validated with corresponding data from experiments or databases such as BacDive (Reimer et al., 2021). If model predictions are insufficient, the process is re-iterated starting with manual curation. Several software packages provide automated pipelines for genome-scale reconstruction (Mendoza et al., 2019; Zimmermann et al., 2021).

The described procedure was developed for isolated and characterized species with sequenced genomes but it can also be applied to MAGs and other metagenomic assemblies (Zimmermann et al., 2021; Zorrilla et al., 2021). The quality of the input genome ultimately determines the quality of the genome-scale reconstruction and it should be noted that MAGs may contain errors or be incomplete (Segata et al., 2013; Frioux et al., 2020). Thereby, reconstructed metabolic networks may contain gaps where certain reactions are missing. Automated gap-filling algorithms are a part of pipelines such as CarveMe and gapseq (Machado et al., 2018; Zimmermann et al., 2021) which generate simulatable reconstructions and have both been applied to build reconstructions from metagenomic sequences (Zimmermann et al., 2021; Zorrilla et al., 2021). Both pipelines utilize a universal metabolic network and extract subnetworks by “carving out” reactions not supported by genomic data. The metaGEM pipeline by Zorrilla et al. (2021) provides a complete workflow to build models from raw metagenomic reads. MetaGEM uses CarveMe and can additionally estimate taxonomic microbiome composition and growth rates. An advantage of using metagenomic sequences is that they represent the current genome of a microbiome member which can be subject to dynamic exchange of genes, for example, by horizontal gene transfer (Zorrilla et al., 2021).

The lack of available data challenges the reconstruction process for microbiome members. During the process, species-specific features such as cofactor usage are included (Thiele and Palsson, 2010) and this information might not be available for uncharacterized species. Another feature added during reconstruction is the biomass reaction which represents biomass synthesis from precursor molecules such as nucleic acids, carbohydrates, lipids, and protein. The stoichiometry of each macromolecule in the biomass reaction is derived experimentally from the macromolecular composition of biomass (Beck et al., 2018; Lachance et al., 2019). Because such data are usually unavailable for microbiome members, biomass reactions from other organisms are adopted (Tobalina et al., 2015; Machado et al., 2018; Zimmermann et al., 2021). However, biomass compositions can differ significantly between organisms and can even depend on growth conditions (Lachance et al., 2019; Sakarika et al., 2023). At the same time, the accuracy of quantitative model predictions depends on the biomass reaction (Gottstein et al., 2016; Lachance et al., 2019). Single-cell and flow cytometry-based techniques could be useful to isolate individual species and determine their macromolecular composition subsequently to create biomass reactions (Cermak et al., 2016; Hatzenpichler et al., 2020). In conclusion, due to the lack of available data, genome-scale reconstructions and resulting constraint-based models of microbiome members are usually not as accurate as models of well-characterized model species such as *Escherichia coli*.

The validation of genome-scale reconstructions is usually done using constraint-based modeling. Model validation can be qualitative [e.g., the model correctly predicts known fermentation products (Zimmermann et al., 2021)] or quantitative [e.g., the model correctly predicts the growth rate on a substrate (Thiele and Palsson, 2010)]. Obtaining suitable data for validation can be challenging for uncharacterized species. Therefore, models of individual species can be assembled into microbiome models (Section 9.2.1) allowing for validation through comparisons between predicted microbiome composition, growth rates, product formation, and substrate utilization, and corresponding data from metaomics. Metabolomics data, for example, can quantify enzyme activities, substrate utilization, fermentation products, and nutrient requirements and can be retrieved *in situ* (Geier et al., 2020). Metaproteomics data could also be utilized for model validation by comparing the occurrence of a metaprotein with the predicted activity of related model reactions or by comparing pathway mappings (Walke et al., 2021) with predicted pathway activities (Li and Figeys, 2020; Rosario et al., 2020).

Instead of using metagenomic sequences for genome reconstruction, it is also possible to map identified species to related available reference reconstructions (Aden et al., 2019; Zorrilla et al., 2021). This can be beneficial to obtain reconstructions of higher quality but might not be representative of the investigated microbiome (Zorrilla et al., 2021). Reference reconstructions are for example available through large-scale reconstruction efforts such as AGORA for species from the human gut microbiome (Magnúsdóttir et al., 2016; Heinken et al., 2023), or from studies like Bernstein et al. (2019), focusing on the human oral microbiome.

The Reconstruction pipelines utilize one (meta)genome to generate a single species genome-scale reconstruction. Microbiome models are typically assembled by treating single-species models as individual compartments connected by a shared medium compartment (Gottstein et al., 2016; Chan et al., 2017; Koch et al., 2019; Diener et al., 2020). The alternative “enzyme-soup” approach merges all reactions and metabolites of different species into one metabolic network. “Enzyme-soup” models have been created from metagenomic and metaproteomic data and used to investigate topological shifts in metabolic networks, active metabolic pathways, and species contributions to metabolic functions (Greenblum et al., 2011; Tobalina et al., 2015). However, these models can only investigate interactions between the microbiome and the environment. Hereafter, analysis methods applied to compartmentalized models are explained.

### 9.2 Constraint-based microbiome modeling

Kinetic parameters for dynamic models of metabolism are difficult to acquire, therefore a steady state is assumed for metabolism, simplifying the system of differential equations into a system of linear algebraic equations (Equation 2) (Gottstein et al., 2016). The steady state assumption applies during microbial growth in continuous cultivation and the exponential phase of batch cultivation (Gottstein et al., 2016). In the steady state, metabolite concentrations are constant over time and thereby only metabolic fluxes can be calculated from the equation system (Equation 2). The unit for reaction rates of biochemical reactions is *mmol*/(*g*_*DW*_*h*) (millimole per gram dry weight per hour) and 1/*h* for the rate of the biomass reaction, i.e., the specific growth rate. A solution of the system is termed flux distribution. For larger networks, the system is under-determined, meaning multiple possible solutions solve Equation (2) creating a solution space (Gottstein et al., 2016).

Flux balance analysis (FBA) is a method, which determines a flux distribution fulfilling an objective and additional constraints. To this end, upper and lower limits for reaction rates are set as constraints (e.g., restriction of oxygen uptake in anaerobic systems) and an objective function is defined. The objective function usually represents a biological objective, for example, biomass growth which is the reaction rate of the biomass function. This is equivalent to maximizing growth yield on the limiting nutrient (Gottstein et al., 2016). The resulting optimization problem can be solved by linear optimization, which determines a global optimum for the objective function (Gottstein et al., 2016). Flux variability analysis (FVA) can be used to explore the limits of the solution space, by performing FBA for each reaction to find its minimal and maximal values (Gudmundsson and Thiele, 2010). The optimization method used in FBA has been extended to determine static and dynamic flux distributions for microbiomes explained in Section 9.2.2. For a complete deviation of the optimization problem from the system of differential equations and further discussion of the limitations of FBA, interested readers are referred to the article by Gottstein et al. (2016).

#### 9.2.1 Simulating steady state metabolic fluxes in microbiomes

Common methods for microbiome FBA utilize compartmentalized microbiome models where each species is treated as an individual compartment and placed in an exchange compartment corresponding to the microbiome medium. Metabolites can be consumed and produced by microbiome members implemented by transport reactions for metabolite transport between medium and species compartments. Additionally, the contribution of biomasses from microbiome members to a total microbiome biomass reaction is implemented to account for microbiome growth. An additional assumption can be introduced stating that in microbiomes with stable compositions, no species can outgrow others, i.e., that growth is balanced. For microbiome FBA, the optimization problem becomes non-linear but can be linearized by fixing either microbiome composition or community growth rate (Khandelwal et al., 2013) (See Khandelwal et al., 2013, Chan et al., 2017, or Koch et al., 2019 for a derivation of the optimization problem).

The optimization problem in microbiome FBA has been addressed by several methods, aiming to identify metabolic fluxes, a microbiome composition, and a microbiome growth rate. The method by Khandelwal et al. (2013), for example, iteratively calculates the maximal microbiome growth rate for different microbiome compositions, until a global maximum for microbiome growth rate is identified. Chan et al. (2017) developed the SteadyCom method, which iteratively maximizes the production of biomass for fixed microbiome growth rates until a maximal microbiome growth rate is determined. The method by Koch et al. (2016) fixes microbiome growth and minimizes a weighted sum of substrate uptakes, which is equivalent to maximizing growth yield.

The advantage of microbiome FBA is that it can be integrated with data. For example, relative abundance data can be directly inserted as microbiome composition, or for microbiomes grown in chemostats, the dilution rate can be set as microbiome growth rate (Gottstein et al., 2016; Koch et al., 2019). Essential metabolic uptakes at maximal microbiome growth can be determined from FVA, indicated by minimal and maximal fluxes having the same sign (Gottstein et al., 2016). Notably, microbiome FBA is subject to the metabolic steady state and balanced growth assumptions, which may only apply in environments with constant conditions such as chemostats (Gottstein et al., 2016). However, with the argument that species abundances in the gut microbiome are on average stable over time, FBA has been applied to gut microbiomes (Chan et al., 2017). Additionally, the assumption of growth maximization may only apply to microbiomes in lab cultures that have evolved toward this objective. Thereby maximal growth rates from FBA should be interpreted as the organism's or microbiome's potential for growth (Gottstein et al., 2016). Furthermore, no regulatory effects are included, no absolute metabolite concentrations can be determined, and model predictions depend on reaction rate constraints (Gottstein et al., 2016).

An exemplary study by De Bernardini et al. (2022) investigated interactions of microbiomes involved in biogas upgrading. The exhaust digestate of biogas fermenters can be fed to bioreactors containing biofilms that fix hydrogen and *CO*_2_ into methane, thus upgrading biogas quality. The authors generated MAGs from biofilms in such bioreactors and created genome-scale reconstruction using gapseq. From the five most dominant MAGs, they created microbiome models and performed microbiome FBA. This gave insight into cross-feeding mechanisms of the microbiome whereby the authors found that most *CO*_2_ is converted to methane via intermediate electron donors such as acetate and found a potential syntrophy based on amino acid exchange.

#### 9.2.2 Simulating dynamic metabolic fluxes in microbiomes

An apparent downside of microbiome FBA is its limitation to steady state predictions. Dynamic FBA (dFBA) inserts FBA into the numerical integration of differential equations for biomass and substrate concentrations, enabling time-course simulations of single and multiple species (Gottstein et al., 2016). This is implemented by calculating the maximal substrate uptake rate in the current time step of one or multiple constraint-based models by Michaelis-Menten kinetics (Equation 1). FBA calculates the growth rates for each model for each numerical integration step, which is then used to determine biomass and substrate concentrations for each following time step (Popp and Centler, 2020). The main assumption of dFBA is that metabolic processes are faster than changes in external concentrations, resulting in cells being in a quasi-steady state before concentration changes occur (Gottstein et al., 2016). Regulatory processes, occurring at slower time scales than metabolic reactions are not considered. Another advantage of dFBA over microbiome FBA is that no community objective is required and simulation of large microbiomes is possible. However, kinetic parameters for substrate uptake need to be provided (Gottstein et al., 2016; Popp and Centler, 2020).

dFBA has been used to simulate growth dynamics and engineering of synthetic communities (Gottstein et al., 2016; Popp and Centler, 2020; García-Jiménez et al., 2021). Lecomte et al. (2024) recently used dFBA to simulate a three-species community for cheese production. They extended the standard version of dFBA by regulation mechanisms for population growth, pH, and selected metabolite exports. The model was calibrated with data from single-species cultures and could be used successfully to predict the dynamics over the seven weeks of the cheese manufacturing process. However, the authors pointed out the necessity of model curation to obtain accurate predictions.

#### 9.2.3 Investigating microbial ecology using genome-scale reconstructions

The availability of reconstruction pipelines and reference reconstructions such as AGORA, facilitate large-scale studies characterizing ecological interactions in microbiomes based on metabolism. Typically, such studies investigate functional redundancy or the prevalent ecological interaction types.

Aden et al. (2019) investigated the microbiome of IBD and rheumatic disease patients during treatment with anti-inflammatory anti-TNF. They acquired taxonomic microbiome profiles from 16s rRNA gene abundance data of fecal samples and collected AGORA reconstructions for detected taxa. For each disease and disease state, they characterized potential types of ecological interactions. This was done by simulating whether growth in pairwise constellations would be higher or lower compared to single-species growth. The authors found no difference in mutualistic interactions compared to controls but noted a reduction of antagonistic interactions at the beginning of therapy in IBD patients. This reduction was restored toward the end of therapy. Furthermore, the authors found increased resource competition in IBD patients which they linked to reduced stability of IBD microbiomes. Furthermore, they simulated the complete microbiome for each sample and found that IBD microbiomes with fewer predicted metabolic interactions might reduce therapeutic success.

Machado et al. (2021) systematically investigated the ratio of cross-feeding and resource competition in thousands of microbiomes across different habitats. They utilized “species metabolic interaction analysis” (SMETANA) (Zelezniak et al., 2015), a method that determines potentials for metabolic interactions and the ratio of overlapping resources as measures for cross-feeding and competition, respectively. The authors found a polarization of cooperative and competitive microbiomes, where cooperative microbiomes showed many auxotrophies, had smaller genomes, and were more often free-living or host-associated. Competitive microbiomes on the other hand had larger genomes with overlapping gene functions, contained many genes related to antimicrobial activity, and were mostly located in soils. Simulations of perturbations showed for cooperative microbiomes a higher susceptibility to species invasion but resilience to nutrient shifts, while the opposite trend was observed for competitive microbiomes. Thereby this study could demonstrate a trade-off between competition and cooperation.

Similarly, Marcelino et al. (2023) performed a meta-study evaluating metabolic interactions in diseased human gut microbiomes. They aimed to identify disease-specific disruptions of metabolite exchanges. The authors reconstructed microbiome models from fecal metagenomes and simulated microbiome growth. Based on microbiome FBA, they determined the capability to exchange metabolites across species for healthy and disease samples. They found important metabolites, such as thiamin and short-chain fatty acid precursors, to be significantly altered between healthy and diseased samples. Furthermore, they predicted metabolites previously shown to be disease-related, including known biomarkers for disease progression. In a case study for Crohn's disease, the authors investigated the causes of altered metabolic exchanges of *H*_2_*S*, which can cause gut inflammation. Resultingly, a disbalance in *H*_2_*S*-producing and consuming species was identified as the origin of altered *H*_2_*S* exchanges.

### 9.3 Contextualized and enzyme-constrained models

Genome-scale reconstructions contain all possible biochemical processes encoded by the genome. However, most processes are subject to gene or post-translational regulation and only active in specific conditions (Feist et al., 2008; Orth et al., 2010). Contextualization adjusts a model to experimental data so that it reflects a specific biological scenario such as a growth condition or a tissue type. Contextualizing a model for growth on a specific substrate, for example, could be done by introducing measured reaction rates and a biomass reaction for this scenario or removing inactive metabolic reactions from the model. Thereby, contextualized models are useful because they are less general and may exclude implausible predictions.

The input for contextualization methods is a constraint-based model, (meta)omics data, information from biochemical databases, and mechanistic knowledge. (Meta)omics data are mapped to model elements and used to knock out (switch-based) not-supported metabolic reactions or constrain them (valve-based) (Hyduke et al., 2013). Contextualization is (semi-)automated and requires annotation of model elements with standard database identifiers to facilitate data mapping.

An example of switch-based contextualization is tINIT (Agren et al., 2014), which scores enzymes and metabolites according to transcriptomic, proteomic, and metabolomics abundance data. Afterwards, it extracts a sub-network that includes reactions supported by the data and excludes reactions with low evidence. Additionally, metabolic functions that should be included in the output model can be specified. The output model contains fewer reactions than the original model.

Enzyme-constrained modeling methods such as GECKO (Domenzain et al., 2022) and sMOMENT (Bekiaris and Klamt, 2020) impose protein allocation constraints on the input model by adding reactions describing the availability of enzymes. Total protein content, absolute proteomic abundances of enzymes, and *k*_*cat*_ values are used to constrain the limits for enzyme usage. In addition to metabolic fluxes, enzyme-constrained models can also predict enzyme usage. Generated output models contain more reactions than the input.

The exemplified methods generate output models in standard formats, that can perform standard analyses, which does not apply to all methods (e.g., Yizhak et al., 2010; Tian and Reed, 2018). More information on contextualization and enzyme-constrained modeling is available in reviews by Opdam et al. (2017); Kerkhoven (2022). The introduced methods are tailored to single-species models and have, to our knowledge, not been applied in microbiome modeling. However, they could be applied to constraint-based models of individual species before assembling them into the microbiome model.

Metatranscriptomic and –proteomic data could be applied to exclude non-expressed metabolic reactions from microbiome member reconstructions. Relatively quantified molecular abundances could be applicable in tINIT-like methods and usable to compare microbiomes across conditions. Creating enzyme-constrained models from metaproteomic data poses some difficulties because the absolute quantification of metaproteins is not reliable. Furthermore, a strategy to handle metaproteins/protein groups that cannot be classified on the species level would be required. Optionally, uniquely identifiable proteins could be used to impose at least some protein constraints. Another problem is the availability of *k*_*cat*_ values. Innovations in machine-learning based *k*_*cat*_ prediction from protein sequences could alleviate this issue (Li et al., 2022). Lastly, model size needs to be considered because microbiomes may contain several hundreds of species. Microbiome models can thus become very large, which can cause long calculation times for analyses (Hädicke and Klamt, 2017; Koch et al., 2019). Enzyme constraints bloat the number of model elements (Bekiaris and Klamt, 2020) and could be less preferential in contrast to tINIT-like methods, which reduce model sizes (Agren et al., 2012, 2014).

### 9.4 Model reduction

A step beyond contextualization is the reduction of genome-scale models to a minimal size while preserving key qualities of the input model (Hädicke and Klamt, 2017). Potential applications of reduced models are, for example, education, tool benchmarking (Orth et al., 2010), kinetic modeling (Hädicke and Klamt, 2017), hybrid modeling (Section 10), construction of microbiome models containing many species (Koch et al., 2019) and model predictive control (Section 11.2).

Erdrich et al. (2015) developed an algorithm that uses a template model, mandatory reactions, metabolites, and phenotypes as input. It removes unprotected model elements in the first step and subsequently compresses the pruned model by lumping together reactions while preserving phenotypes of the template. Another approach by Koch et al. (2019) reduces compartmentalized community models. The authors first determined conversions of microbial substrates to products (net conversions) for single species models and reduced these models to exclusively represent these conversions. The reduced models were then assembled into a microbiome model and can be utilized to analyze species interactions and microbiome composition.

### 9.5 Benefits and limitations of constraint-based microbiome models

Genome-scale metabolic reconstructions are highly valuable because they serve as knowledgebases that can be refined, extended, and integrated with (meta)omics data, with only an annotated genome required as minimal input (Robinson et al., 2020). Even if the resulting constraint-based models are not refined, they can still be utilized for qualitative predictions. However, with refinement, these models have the potential to provide accurate quantitative predictions. Furthermore, modelers can benefit from available high-quality reconstructions or large-scale collections such as AGORA. Constraint-based models can predict metabolic fluxes in microbiomes without requiring kinetic parameters. Many resources and methods for model analysis are available resulting in a large variety of model applications.

Generating high-quality reconstructions demands significant effort and data, often taking months to years until useful models for quantitative predictions become available (Orth et al., 2010). Compiling a microbiome model can be difficult due to the use of different namespaces for model elements and integration of omics data is impeded by lacking model annotations (Section 12). Furthermore, microbiome FBA is subject to several assumptions such as the metabolic steady state, balanced growth, and an objective, which may not apply to every biological system. Dynamic FBA is independent of a microbiome objective but requires kinetic parameters. Furthermore, the accuracy of FBA predictions depends on reaction rate constraints, which can be set according to maximal uptake rates or ATP maintenance parameters determined experimentally. When such data are unavailable for microbiome members, predictions of microbiome models may be less accurate. Moreover, no regulatory effects are incorporated in standard constraint-based models.

## 10 Combined model frameworks and agent-based modeling

Every modeling framework introduced so far assumes homogeneous populations of organisms or well-mixed systems and is dedicated to modeling one particular biological system. Thereby, to model the interaction of different systems or different scales, a connection of model formalisms is required, also known as hybrid models (Bardini et al., 2017). Essentially, the previously mentioned dFBA is a type of hybrid model as it connects differential equations with constraint-based modeling.

Agent-based modeling (ABM) is a distinct modeling framework that can account for population and spatial heterogeneity. However, it is also included in this section because agent-based models often combine frameworks such as dynamic models for biomass and molecule transport with constraint-based metabolic models. Hereafter some examples for combined modeling frameworks and ABM are shown but further explanations and examples can be found in reviews by Qu et al. (2011), Kreft et al. (2017), Kumar et al. (2019), García-Jiménez et al. (2021), and Liu (2023). A list of software for multi-scale and agent-based modeling is provided in Table 7.

**Table 7:** List of software for agent-based and multi-scale modeling.

**Software (implementation)**	**Application**	**References**
BacArena (R)	Agent-based modeling utilizing constraint-based metabolic modeling	Bauer et al., 2017
COMETS (desktop application/ Python/ MATLAB/ command line)	Agent-based modeling utilizing constraint-based metabolic modeling	Dukovski et al., 2021
IndiMESH (MATLAB)	Agent-based modeling specialized to soil ecosystems	Borer et al., 2019
Morpheus (desktop application)	Modeling and simulation of multi-cellular and multi-scale systems	Starruß et al., 2014

### 10.1 Combining model frameworks connects different cellular systems and spatial scales

Models such as ecological models can represent interactions, but they lack the capability to dissect the mechanisms underlying these interactions. In contrast, constraint-based metabolic models can describe metabolic interactions but typically do not account for signaling and regulation. Therefore, there is a need for models that integrate these mechanisms to fully understand microbiomes, as well as interactions with their hosts.

Genome-scale metabolic reconstructions can implement transcriptional regulation through gene-protein-reaction rules, which are Boolean expressions encoding the genes required for a metabolic reaction to occur. This feature facilitates knock-out studies or the integration of proteomics data with models (Orth et al., 2010; Bekiaris and Klamt, 2020; Filippo et al., 2021; Domenzain et al., 2022). Whole-cell models aim to capture every cellular process but have only been realized for *Mycoplasma genitalium* and *E. coli* (Sun et al., 2021). The *E. coli* whole-cell model, for example, integrates differential equations, constraint-based modeling, and stochastic simulations (Sun et al., 2021).

Another motivation to combine modeling frameworks is the integration of experimental data from different scales (Qu et al., 2011; Lui et al., 2021) and dissecting the influence of molecular mechanisms on dynamics at higher spatial scales. Thiele et al. (2017), for example, discussed the connection of metabolic models with physiologically based pharmacokinetic (PBPK) models. PBPK models are ordinary differential equation models employed to evaluate the dynamics of drug concentration in the human body. These models can be connected with constraint-based models of individual organ and microbiome metabolism. This integration enables the investigation of the involved molecular mechanisms and allows for the incorporation of data on diet and patient-specific information, thereby facilitating personalized drug development (Thiele et al., 2017).

Multi-scale modeling has also been applied to the biogas process. Weinrich et al. (2019) extended the ADM1 model with genome-scale metabolic models of methanogenic (i.e., biogas-producing) microorganisms. The resulting model reproduced simulations of the standard AMD1 model and predicted cellular metabolic fluxes. Weinrich et al. (2019) proposed that such models will facilitate the integration and interpretation of time-resolved metaomics data from biogas plants, estimate process yields, determine interventions for process optimization, and identify signals indicating reactor breakdowns.

The development of multi-scale models is context-specific and thereby modelers usually need to assemble such models by themselves. Lui et al. (2021) developed a conceptual framework for the development of microbiome models spanning scales from genes to ecosystems. Their framework accounts for biotic and abiotic processes such as the transport of strains, growth, direct microbial interactions, mutations, and dynamics of available chemical compounds. It is designed to uncover knowledge gaps, can be streamlined to focus on specific terms of interest, aids in experiment design, and is intended to undergo iterative cycles of parameterization through experimentation across different scales.

### 10.2 Agent-based modeling

Agent-based models, also known as individual-based models, explicitly represent individuals and their behavior in space and time, allowing for the consideration of individuality and resulting heterogeneity within microbial populations (Kreft et al., 2017). The general principle of implementation involves assigning each individual a model representing metabolism (or other processes), along with defined rules for microbial behavior such as cellular motion, division, or death rates (Dukovski et al., 2021). Typically, space is discretely implemented as a two-dimensional grid, with each individual placed in a designated grid cell (Bauer et al., 2017; Dukovski et al., 2021). Additionally, ambient concentrations of compounds are included, including their transport or fluctuation (Bauer et al., 2017; Dukovski et al., 2021).

Agent-based modeling software such as COMETS (Dukovski et al., 2021), is capable of simulating evolutionary processes, growth at soil air interfaces, or the morphology of bacterial colonies. Borer et al. (2022) recently used agent-based modeling to simulate microbial growth in pore networks of soil around carbon source hot spots. They found that growth near the hot spot reduces available oxygen, thereby generating niches occupied by different species.

### 10.3 Benefits and limitations of combined model formalisms

Hybrid and multi-scale models can connect mechanisms and data from different biological systems and spatial scales. Moreover, they are not confined to any specific model framework. Agent-based models stand out for their ability to account for cellular heterogeneity, a feature not inferred from other model types.

A higher mechanistic model resolution results in more kinetic parameters that need to be estimated. Parameter estimation can become more complex because individual model types may need to be calibrated individually or in combination. This makes hybrid and agent-based models computationally more expensive. To reduce the computational burden, agent-based models often utilize coarse-grained models for processes in individual cells (Kreft et al., 2017; Borer et al., 2019).

## 11 How modeling guides microbiome control

Control refers to the regulation of a dynamic system to achieve a desired dynamic behavior. Interventions to control a system are termed control strategies and can be applied to steer the behavior of microbiomes and leverage microbiome models. This section briefly introduces the concept of closed-loop control, discusses elements of closed-loop control concerning microbiomes, and emphasizes model-based control strategies for microbiomes with examples from biotechnology and the human gut. Further information on this topic can be found in the reviews by Lee and Steel (2022) and Liu (2023).

### 11.1 The concept of closed-loop control

**Figure 4:**
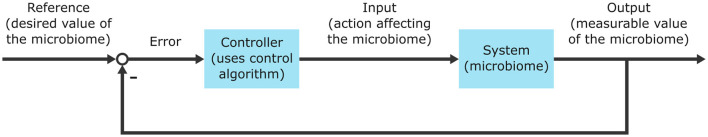
Block diagram of a closed-loop control with feedback. The controller computes an action that affects the microbiome based on a control algorithm. The action is applied to the microbiome, which reacts with a measurable output. The output is compared with the desired reference value. The difference between both values is the error, which is fed back into the controller.

Control strategies can follow a feedback structure (Figure 4) allowing it to affect a dynamic system, such as a microbiome. The system has a measurable output that should be controlled, for example, the concentration of a metabolite. The output response is affected by the system input, for example, the concentration of a specific nutrient. As the system is dynamic, the output may change over time. To validate that the output has a desired value, it is compared regularly to a reference value. The difference between the measured output and the reference is the error. The error is fed back into a controller, which computes a system input according to a control algorithm. The controller tries to maintain a low error. If the error increases, the controller steers the system input to reduce the mismatch between output and reference. Because the controller closes the loop to the system, this feedback structure is named closed-loop control.

#### 11.1.1 System inputs and system outputs of microbiomes

Nutrient concentration was a previous example of an input for a microbiome, but any environmental factor can be altered to influence microbiome output. This includes pH, level of oxygen, temperature, or salinity. Additionally, population sizes of individual species can be targeted by the input (Liu, 2023). Population size can be increased by the expression of growth-inducing genes (Gutiérrez Mena et al., 2022) or by directly adding a species to the microbiome (Aditya et al., 2021; Liu, 2023). On the other hand, the population size can be decreased by introducing bacteriostatics, antibiotics, or targeted bacteriophages (Lu and Collins, 2007; Liu, 2023).

The control output is the response of the system to the input. Several methods exist to measure the output depending on factors such as the complexity of the community, the control goal, the measurement frequency, the economic cost, or the duration of measurements. Process parameters such as the pH value or oxygen concentration are easy, cheap, and quick to measure but do not give any insight into the microbiome. Other methods that are applicable on-line (i.e., “during cultivation”) are flow cytometry or metabolomics. Flow cytometry can distinguish different strains using universal dyes, thereby giving an insight into microbiome composition (Buysschaert et al., 2017). Gas chromatography-based metabolomics can be applied to measure gaseous metabolites during cultivation (Khesali Aghtaei et al., 2022). Metaproteomics in contrast is less suited for on-line measurements due to the extensive sample preparation but can resolve expressed enzymes.

### 11.2 Control algorithms and model-predictive control

The control algorithm determines how the controller steers the system inputs. The selection of the control algorithm depends on the system and the control goals. One of the most straightforward approaches is PID (proportional, integral, and derivative) control. A PID controller consists of three adjustable parameters for corrections based on the proportional, the integral, and the derivative term of the error value. Due to its simple structure, PID control is easy to implement without much knowledge of the system. However, the performance of the controller depends on the chosen parameters. Controller parameters can be tuned using a mathematical model of the system. This results in a more accurate parameter set without the need for extensive experiments. Bensmann et al. (2014), for example, performed a comprehensive simulation study of biogas plants. They used an extended version of the ADM1 model to propose and test a PI (i.e., PID without the derivative term) feed-back control for the biological methanation of hydrogen.

Model predictive control (MPC) is an advanced control strategy for complex control goals or cases where multiple inputs need to be controlled. MPC is an optimal control strategy and, therefore, aims to optimize a given objective function, such as the taxonomic microbiome composition (Liu, 2023). For optimization, MPC uses a model of the system to predict the future system behavior over a finite time horizon. Xue et al. (2015), for example, used nonlinear MPC to control the anaerobic digestion process in biogas plants, employing a reduced version of the AMD1 model. Because many state variables of the anaerobic digestion process are immeasurable, these values need to be estimated. To this end, the authors applied an estimation algorithm termed Unscented Kalman Filtering, which determines parameters based on available measurements (Simon, 2001; Xue et al., 2015).

MPC has also been applied in cybergenetic control. Cybergenetics regulates gene activity in genetically engineered microorganisms by external stimuli, such as light, to control metabolic functions or growth. Espinel-Ríos et al. (2023a) performed cybergenetic simulation studies in which they optimized nianigrin production in a co-culture of engineered *E. coli* and yeast. The same authors implemented cybergenetic MPC for a lactate-producing *E. coli* culture in a bioreactor (Espinel-Ríos et al., 2023b). Here, a dynamic constraint-based model with protein resource allocation was used to control the expression of ATPase by light. This approach could also be extended to synthetic microbiomes, as stated by the authors. Wagner and Schlüter (2020) applied a machine-learning based surrogate model as MPC to control methane production in the biogas process. The model was trained on simulated data from the ADM1 model and could accomplish similar precision as the ADM1 model. The authors applied this procedure to circumvent numerical issues in simulating the ADM1 model.

Recently, Angulo et al. (2019) developed an approach to identify species that could be targeted by control inputs to regulate native microbiomes, such as those in the human gut. Such approaches could enable targeted interventions to guide microbiomes toward a desired composition (Liu, 2023). The approach employs graph theory to identify ”driver species” capable of propagating control inputs throughout ecological networks (Angulo et al., 2019; Liu, 2023). Angulo et al. (2019) applied this concept in a simulation study to regulate the model output of mouse gut and sea sponge microbiomes using linear MPC based on the gLV model (Section 8.1). This approach could even be implemented by applying pulsed inputs at discrete time points and utilizing discontinuously measured data, promising therapeutic potential (Liu, 2023).

## 12 Microbiome modeling requires standards, software, and repositories

Standards facilitate the reuse of data, models, and simulation results. This section describes the concept of FAIR (findable, accessible, interoperable, and reusable) guidelines for research data and expands to the standards of the modeling community. Furthermore, repositories used in the modeling domain are introduced. More information on standards in systems biology is given in articles by Waltemath and Wolkenhauer (2016) and Stanford et al. (2019).

### 12.1 FAIR data

Biological data are generated at a high pace and good data management is required to facilitate the reuse and integration of data. In 2016, the FAIR guidelines were published to improve existing issues in research data management and stewardship (Wilkinson et al., 2016). These principles apply to research data, as well as algorithms, software, and workflows (Wilkinson et al., 2016). Additionally, FAIR guidelines apply to metadata, which is information associated with the “actual” data or software. Metadata describes, for example, the subject of research, data origin, or time of generation. Finding, retrieving, and integrating big amounts of data, for example, to build genome-scale metabolic models requires automation. Hence, another motivation for having FAIR data and software is to provide minimal requirements facilitating automation.

Four main principles are covered by FAIR (explanations are taken from Boeckhout et al., 2018):

- Findability (“Datasets should be described, identified and registered or indexed in a clear and unequivocal manner”).
- Accessibility (“Datasets should be accessible through a clearly defined access procedure, ideally using automated means. Metadata should always remain accessible”).
- Interoperability (“Data and metadata are conceptualized, expressed and structured using common, published standards”).
- Reusability (“Characteristics of data and their provenance are described in detail according to domain-relevant community standards, with clear and accessible conditions for use”).

FAIR is highly relevant for research, but factors such as incomplete metadata and insufficient reporting of parameters and initial conditions hamper the reusability of biological and biomedical data (Hughes et al., 2023) or computational models (Tiwari et al., 2021).

FAIRDOM (https://fair-dom.org/about) is a consortium supporting scientific communities in implementing FAIR guidelines. They provide FAIRDOMHub (Wolstencroft et al., 2016), a web-based repository to publish scientific data, protocols, and models, as well as FAIRsharing (https://fairsharing.org/), a web tool for searching community guidelines and scientific databases.

### 12.2 Initiatives and community guidelines

While FAIRDOM is a more general consortium, the COmputational Modeling in BIology Network (COMBINE) is an initiative establishing standards on the level of the modeling community (Hucka et al., 2015; Waltemath et al., 2020). COMBINE coordinates standards for exchange formats and modeling languages (e.g., systems biology markup language, see below) and organizes regular community meetings (Hucka et al., 2015). Another initiative cooperating with COMBINE is the Consortium for Logical Models and Tools (CoLoMoTo) (Naldi et al., 2015). CoLoMoTo has similar aims as COMBINE but specializes in logical modeling (including Boolean modeling).

COMBINE supports guidelines for metadata on model elements and simulation experiments. Model elements usually represent biological entities or relations between them (e.g., in chemical formulas) and their meaning can be described with metadata. Metadata links model entities to unique identifiers for biological entities. The association of model entities and metadata is termed model annotation, which is important for omics data integration (Novère et al., 2005; Tatka et al., 2023). MIRIAM (Minimum information requested in the annotation of biochemical models) provides guidelines for these annotations aiming to improve model reusability. It specifies model documentation, correspondence between models and articles, utilization of machine-readable exchange formats, and the quality of model annotations (Novère et al., 2005).

MIASE (Minimum Information About a Simulation Experiment) is complementary to MIRIAM and provides guidelines facilitating the reproduction of simulation experiments (Waltemath et al., 2011). MIASE-compliant reporting includes the specification and definition of used models, precise descriptions of simulation steps, and descriptions of the analysis of simulation data (e.g., post-processing steps) (Waltemath et al., 2011).

### 12.3 Languages for modeling and exchange formats

The interoperability principle in FAIR specifies the use of formal languages to express knowledge (Wilkinson et al., 2016). Systems biology has adopted this principle to describe model structures and simulation experiments.

The systems biology markup language (SBML) is a widely used standard in the metabolic modeling community (Carey et al., 2020) and one of the languages maintained by COMBINE. It builds on the extensible markup language (XML) and describes model structures while being agnostic to any software or analysis method (Hucka et al., 2019). A constraint-based metabolic model, for example, is represented by semantic elements describing biological entities (reactions, metabolites, gene products, and compartments) and default parameters. These semantic elements are organized hierarchically, and specific information is assigned by element attributes. An important aspect of SBML is the use of systems biology ontology (SBO) terms to characterize model elements (e.g., mathematical expressions, metadata, or physical entities) (Hucka et al., 2019).

SBML is a modeling language and exchange file format at the same time. Furthermore, it allows the implementation of MIRIAM guidelines by providing means for model annotation, fostering the reusability of models. For annotation, the resource description framework (RDF) is utilized, supporting references to multiple (biochemical) databases (Hucka et al., 2019). Additionally, the current SBML version 3 is designed in a modular manner, providing extensions to the core language for the representation of constraint-based, ODE, and Boolean models, as well as means to store network layout information (Keating et al., 2020). A software package/application aiding the implementation of MIRIAM guidelines in genome-scale metabolic models is MEMOTE (Lieven et al., 2020). MEMOTE facilitates quality control for annotations and model consistency and provides a framework to set up version-controlled repositories for model development.

SED-ML is another important XML-based format to describe simulation experiments. SED-ML is maintained by COMBINE and compliant with MIASE. More information can be found in articles by Köhn and Novère (2008); Hucka et al. (2015).

### 12.4 Repositories

Repositories are platforms to store and share data or models. They are accessible through websites or programmatically via application programming interfaces (API). Repositories for biochemical and experimental data are vital to annotate metaomics data (Section 4.2) but also essential for network reconstruction, validation, refinement, and contextualization of models. A list of biochemical databases for model annotation can be found in the supplementary material of Lieven et al. (2020). Other resources can be found on the FAIRSharing platform, which indexes domain-specific databases, for example, STRING for PPI networks (Szklarczyk et al., 2020), BacDive for growth screenings (Reimer et al., 2021), Sabio-RK (Wittig et al., 2017), and BRENDA (Chang et al., 2020) for enzyme constants or MGnify for microbiome sequence analysis and storage (Mitchell et al., 2019).

Models are published in dedicated repositories or on GitHub (e.g., https://github.com/SysBioChalmers/Human-GEM), an online platform for version-controlled projects commonly used in software development. BioModels is one of the biggest dedicated model repositories. It contains different model types, models are partly curated and provides a version control system (Malik-Sheriff et al., 2019). BiGG is a fully curated repository providing constraint-based models and model elements (King et al., 2015). Model elements are aligned to a common namespace (i.e., a naming scheme) and contain cross-references to biochemical databases. MetaNetX is another database for constraint-based models, which collects its entries from various resources (including BiGG) and aims to unify models under the MNXref namespace (Moretti et al., 2020).

The list of explicit microbiome models in public repositories is short. Except for BioModels, all mentioned model repositories contain single-species models. Using the keywords “microbiome” and “microbial community” in BioModels resulted in six models representing more than one species (date of access: August 4, 2023, Supplementary Table S2). However, a common strategy for metabolic models is to make models of individual species available and share the code to assemble microbiome models, as done, for example, by Ankrah et al. (2021) and Heinken et al. (2023).

### 12.5 Remarks on languages and software for community modeling

Even though several initiatives and standards are set up, modeling is not FAIR. A survey among 89 members of the constraint-based modeling community showed that only 56% were aware of MIRIAM (Carey et al., 2020), which is in accordance with Lieven et al. (2020), who demonstrated that many constraint-based models lack annotation or semantic SBO identifiers. MIASE was familiar to less than 25% of constraint-based modelers, pointing out potential issues in reporting simulation experiments. This hypothesis applies at least to kinetic models, as shown by Tiwari et al. (2021). They tried to reproduce 455 kinetic models from the BioModels repository, which was possible for only 49% based on information from respective publications. The main reasons for irreproducibility were inconsistencies in model structure, as well as insufficient reporting of initial values and parameters.

Kim et al. (2018) showed that irreproducibility also occurs for bioinformatics software: Conflicts of operating systems, dependency issues, and poor documentation are common examples researchers must face when using foreign code (Kim et al., 2018). Additionally, researchers without advanced training in programming or bioinformatics will quickly surrender, as resolving these issues requires some debugging experience. A resolution to this issue could be the use of lightweight software containers (Boettiger, 2015). Such containers are isolated from the hosting system and run their own operating system, preinstalled dependencies, and configurations, allowing to share containerized software (https://docs.docker.com/get-started/) (Boettiger, 2015). Naldi et al. (2018), for example, implemented a containerized environment for several software packages for Boolean modeling.

Reusability ultimately affects microbiome modeling, because microbiome models can consist of individual sub-models (from third parties) that need to be reusable. Even if sub-models are annotated, identifiers for biological entities can be ambiguous (Pham et al., 2019). Furthermore, there is no standard namespace for model elements, and merging models from different sources can be problematic if no common identifiers or annotations are included (i.e., if the models use different namespaces) (Chindelevitch et al., 2012). To alleviate this problem, MNXref aims to provide a common namespace by connecting several database references to unique identifiers usable for model annotation (Moretti et al., 2020).

Based on the recommendations for constraint-based model annotation provided by Ravikrishnan and Raman (2015), the identifiers tested by MEMOTE (Supplementary Table S3) (Lieven et al., 2020), and own experience, the recommended set of identifiers for minimal annotation includes:

- **All model elements:** SBO identifiers (Hucka et al., 2019).
- **Reactions:** EC numbers, MNXref.
- **Metabolites:** sum formula, key from a biochemical database [e.g., InChI (Goodman et al., 2021), ChEBI (Hastings et al., 2015), KEGG (Kanehisa et al., 2022)], MNXref.
- **Genes:** UniProt Accession (Bateman et al., 2022).

For each species included in a model or in models representing individual species, the NCBI or GTDB taxonomy (Schoch et al., 2020; Parks et al., 2021) should be included as well.

(Meta)omics data should include the respective identifiers to facilitate data integration. Following the suggested set of minimal annotations, metabolomic data should include InChI, ChEBI, and MNXref identifiers, and genomic, transcriptomic, or proteomic data should include EC numbers, MNXref identifiers, and UniProt Accessions.

Carey et al. (2020) pointed out that community standards are inherently lagging behind new analysis methods. This could also be a reason that most available genome-scale community models need to be assembled from their member species and require the original code to assemble microbiome models. Nevertheless, SBML can represent compartmentalized metabolic community models, but there is still a lack of standards for other model types, e.g., agent-based models (Vieira and Laubenbacher, 2022). A future solution could be the addition of new SBML extensions to keep up (Carey et al., 2020).

Prospectively, it will take further time and effort to assimilate guidelines into the modeling community and minimize reproducibility issues. Giving more incentives by rewarding model annotation, stricter requirements by journals, providing user-friendly annotation tools, peer-reviewing models and software, and coordinating standardization efforts are examples of potential large-scale solutions to the problem (Carey et al., 2020; Papin et al., 2020; Tiwari et al., 2021; Hughes et al., 2023).

## 13 Discussion

The holistic approach of systems biology paves the way to understanding microbiomes. Every aspect of systems biology, i.e., measuring metaomics data, data integration, data analysis, and modeling is linked with a vast amount of challenges and options. Only specialists can overview the challenges and options in their research area. At the same time, it is counterproductive to study them in isolation from other areas. This review aims to contribute to dissolving the barrier toward microbiome modeling and provides directions for further self-education.

The isolation and characterization of new species play a crucial role for microbiome modeling. Data on individual species, such as growth phenotypes and genome sequences, are invaluable for assessing the potential for ecological interactions of organisms. Pure cultures are also essential for determining model parameters for individual organisms, such as biomass composition or maximal uptake rates. Such data are vital for building high-quality single-species models, which can then be utilized to construct microbiome models, as discussed in Section 9. Furthermore, time-course data of individual strains can be utilized to estimate parameters in microbiome models, as demonstrated by Venturelli et al. (2018). Available strains can also be used to cultivate reduced or synthetic microbiomes, which are essential for validating microbiome models (García-Jiménez et al., 2021). While control strategies for biotechnological processes, such as the biogas process, are widely implemented, they are only now becoming available for human gut microbiomes and will require model systems based on cultivated microbiomes for testing before they can be realized in patients (Liu, 2023).

Improvements in metaomics methods and technology will provide standardized workflows and more reliable data (Heyer et al., 2017; Arıkan and Muth, 2023; Wolf et al., 2023), which will also benefit microbiome modeling. For instance, higher-quality MAGs could be provided for genome-scale metabolic reconstructions, or better-resolved metaproteomics data could be utilized to create contextualized or enzyme-constrained microbiome models. Such microbiome models are suitable for studies investigating microbiome ecology such as those presented by Aden et al. (2019), Machado et al. (2021), and Marcelino et al. (2023), as this would result in more realistic predictions of ecological interactions. Other technologies, such as non-destructive methods and methods based on flow cytometry could be applied more frequently to probe active species and provide a better separation of taxa for downstream omics analyses and isolated cultivation (Hatzenpichler et al., 2020). These technologies can also resolve population heterogeneity which could be integrated into agent-based models. Multiomics applied to microbiomes is also promising, as it will provide multiple molecular layers for model building and validation and could be integrated into hybrid or whole-cell microbiome models.

New bioinformatics methods will also increase the amount of information extractable from metaomics data. For example, unknown enzymes can be uncovered and functionally characterized from metaomics data (Jia et al., 2022). Previously unknown enzymatic reactions can be introduced into microbiome models, such as molecular interaction graphs or constraint-based models, to evaluate the role of such previously unknown enzymes in ecological interactions. A recent study by Li et al. (2022) utilized machine learning to predict *k*_*cat*_ values for enzymes from substrate structures and protein sequences. They used these predicted values to create enzyme-constrained metabolic models (Section 9.3) that achieved better prediction results than enzyme-constrained models created with previous pipelines. Potentially such approaches could aid the parametrization of microbiome models even if included enzyme parameters have not been characterized.

The most common microbiome modeling frameworks were presented, yet none of them is perfect. Models are subject to assumptions that may not always apply, mechanistic and spatial resolution are limited, models can depend on many parameters, and sometimes only qualitative predictions can be made. Such disadvantages could be counteracted by combining different modeling frameworks, as demonstrated by Steinway et al. (2015). Another example is hybrid modeling using machine learning, as employed by Espinel-Ríos et al. (2023a). Their hybrid model consists of a dynamic mechanistic model coupled to a neural network, which predicts uncertain variables of the mechanistic model. Such approaches could be applied where parts of a mechanism in a microbiome are unknown, but sufficient training data are available.

Furthermore, other frameworks could be explored further for microbiome modeling, such as Petri nets which have been utilized for modeling the spread of antibiotic resistance in microbiomes (Bardini et al., 2018). Rule-based modeling is another formalism promising the genome-scale modeling of signaling and regulation (Romers et al., 2020). Rule-based models could be used to create models of host signaling and regulation coupled with microbiome models to investigate the molecular interactions of microbiomes and hosts. Efforts in this direction are underway as microbiome models have already been coupled with dynamic models of human organ systems (Thiele et al., 2017). The microbiome has also been included in a metabolic whole-body model of humans containing more than 80,000 metabolic reactions (Thiele et al., 2020). Model reduction techniques (discussed in Section 8.2 and Section 9.4) will become very useful in reducing the computation times of such complex models.

Despite its utility in providing mechanistic understanding and controlling microbiomes, microbiome modeling is not fully established in the standard workflow of metaomics data analysis. A potential reason for this could be the lack of accessibility as microbiome modeling mostly relies on bioinformatics experience. Furthermore, there is a lack of standardization even in bioinformatics workflows for metaomics data analysis, which is slowly counteracted by initiatives and ring trials such as CAMPI3 (https://metaproteomics.org/campi/campi3/). The cooperation of lab experts and bioinformaticians/modelers is one solution to establishing modeling and has already been realized by many research groups. The second option is to provide user-friendly software for microbiome modeling, such as KBase (Arkin et al., 2018). A drawback of such software is that it takes time to implement new features. For example, KBase is focused on processing genomic data but has limited features for handling metaproteomic data or for microbiome model analysis.

The realization of guidelines such as FAIR facilitates a landscape of data and model repositories and available software for microbiome modeling. Nevertheless, standards are not fully established in modeling communities and many are unaware of their existence. As a result, many models are not reusable for data integration because of missing or not unified annotations and simulation results are not reproducible. In addition, standards naturally are behind emerging analysis methods, whereby it is often the case that original code from publications needs to be executed. However, software is affected by irreproducibility as well. Containerizing software for modeling or implementing web applications are short-term perspectives to make microbiome modeling accessible for researchers. In the long run, standards need to be assimilated by scientific communities, which could be facilitated by repositories and journals giving incentives for the usage of standards, as well as peer-reviewing of models and software.

## Author contributions

EL: Conceptualization, Data curation, Investigation, Methodology, Project administration, Software, Writing – original draft, Writing – review & editing. LK: Investigation, Writing – review & editing, Writing – original draft. JK: Conceptualization, Writing – review & editing. DB: Conceptualization, Writing – review & editing. RH: Conceptualization, Investigation, Project administration, Supervision, Writing – original draft, Writing – review & editing.

## Footnotes

^1^With the term “microbiomes,” we include the terms “microbial community,” or “microbiota” and refer to any community of microorganisms living with or without a eukaryotic host.

^2^https://www.destatis.de/DE/Presse/Pressemitteilungen/2023/03/PD23_090_43312.html (accessed December 13, 2023).

^3^https://www.umweltbundesamt.de/daten/energie/erneuerbare-energien-vermiedene-treibhausgase#stromerzeugung, (accessed December 13, 2023).

^4^ https://www.connectedpapers.com/
