# Supplementary material for: Microbiome modeling: a beginner's guide
Source: Front Microbiol. 2024 Jun 19;15:1368377. doi: 10.3389/fmicb.2024.1368377 (PMC11220171; doi:10.3389/fmicb.2024.1368377)
Supplement: Supplementary file 1 [file Data_Sheet_1.zip › supplementary.pdf]

Supplementary Material

1 TARGETED LITERATURE RESEARCH STRATEGY DATA

To extract relevant papers on the covered topics, we used a Python script to query the PubMed API (Sayers, 2009). The script retrieves an initial list of articles for a query, indexes and counts the occurrence of all references of these initial articles. We performed a search for every section or larger subsection (table 1). The resulting Excel files are listed below (table S1) and are available in the supplementary files of this manuscript. The source code and all output files are available on our GitHub repository (`https://github.com/voidsailor/targeted_literature_search`) under MIT license.

Table S1. Pubmed query results.

file number	file name
1	07_11_23_microbiome_or_microbial_community_100_list.xlsx
2	07_11_23_meta_proteomics_or_meta_genomics_or_meta_omics_100_list.xlsx
3	07_11_23_computational_modeling_and_metabolism_or_regulation_or_signaling_100_list.xlsx
4	07_11_23_biological_network_reconstruction_and_microbiome_or_microbial_community_100_list.xlsx
5	07_11_23_computational_modeling_and_parameter_estimation_or_contextualization_or_reduction_100_list.xlsx
6	07_11_23_computational_modeling_and_microbiome_or_microbial_community_100_list.xlsx
7	07_11_23_control_algorithm_and_microbiome_or_microbial_community_100_list.xlsx
8	07_11_23_network_modeling_and_guidelines_or_software_or_repository_100_list.xlsx

2 MICROBIOME MODELING REQUIRES STANDARDS, SOFTWARE AND REPOSITORIES

A search in the model databases BiGG, MetaNetX, and BioModels for models containing more than one species resulted in six hits (table S2, date of access: August 4, 2023). All of these hits were found in BioModels using ”microbiome” and ”microbial community” as queries.

For the integration of (meta)omics data, annotations of model elements such as genes, proteins, reactions, and metabolites are required. MEMOTE is a software for genome-scale metabolic model testing (Lieven et al., 2020) and tests for a list of standard databases (table S3). This list could be used as a reference for identifiers that should be included in genome-scale models and (meta)omics data.

REFERENCES

Lieven, C., Beber, M. E., Olivier, B. G., Bergmann, F. T., Ataman, M., Babaei, P., et al. (2020). MEMOTE for standardized genome-scale metabolic model testing. *Nature Biotechnology* 38, 272–276. doi:10.1038/s41587-020-0446-y

Sayers, E. (2009). Entrez programming utilities help. *URL http://www.ncbi.nlm.nih.gov/books/NBK25499* 17

Table S2. Microbiome models in public model repositories.

repository	model id	model name	species	model type
BioModels	MODEL2106100001	Konstantinidis2021 - Persistence of mutants in a mutualistic microbial co-culture	Saccharomyces cerevisiae, Lactobacillus plantarum	Individual-based
BioModels	MODEL2209060002	Mostolizadeh2022 - D. pigrum and S. aureus	Staphylococcus aureus, Dolosigranulum pigrum	constraint-based
BioModels	BIOMD0000000583	Leber2015 - Mucosal immunity and gut microbiome interaction during C. difficile infection	Clostridioides difficile, Mus musculus	kinetic
BioModels	BIOMD0000000625	Leber2016 - Expanded model of Tfh-Tfr differentiation - Helicobacter pylori infection	Helicobacter pylori, Mus musculus	kinetic
BioModels	MODEL2002070001	Geißert2020 - Yersinia enterocolitica co-infection in mice	Yersinia enterocolitica, Mus musculus	kinetic
BioModels	BIOMD0000000596	Philipson2015 - Innate immune response modulated by NLRX1	Helicobacter pylori, Mus musculus	kinetic

Table S3. Database identifiers tested in MEMOTE. The list was adapted from the annotation.py file in MEMOTE's GitHub repository `https://github.com/opencobra/memote/blob/develop/src/memote/support/annotation.py`.

database identifier	model component	url
MetaNetX	reactions, metabolites	http://www.metanetx.org
Kegg	reactions, metabolites, genes/proteins	http://www.kegg.jp/
SEED	metabolites	http://modelseed.org/
InChI	metabolites	https://www.ebi.ac.uk/chebi/
InChIKey	metabolites	http://cactus.nci.nih.gov/chemical/structure
ChEBI	metabolites	http://bioportal.bioontology.org/ontologies/CHEBI
BRENDA	reactions	http://www.brenda-enzymes.org/
RHEA	reactions	http://www.rhea-db.org/
HMDB	metabolites	http://www.hmdb.ca/
BioCyc	reactions, metabolites	http://biocyc.org
Reactome	reactions, metabolites	http://www.reactome.org/
BiGG	reactions, metabolites	http://bigg.ucsd.edu/universal/
PubChem	metabolites	https://pubchem.ncbi.nlm.nih.gov/
RefSeq	genes/proteins	http://www.ncbi.nlm.nih.gov/projects/RefSeq/
Uniprot	genes/proteins	http://www.uniprot.org/
EC-Code	reactions	http://www.enzyme-database.org/
EcoGene	genes/proteins	http://ecogene.org/
NCBI GI	genes/proteins	http://www.ncbi.nlm.nih.gov/protein/
NCBI Gene	genes/proteins	http://ncbigene.bio2rdf.org/fct
NCBI Protein	genes/proteins	http://www.ncbi.nlm.nih.gov/protein
CCDS	genes/proteins	http://www.ncbi.nlm.nih.gov/CCDS/
HPRD	genes/proteins	http://www.hprd.org/
ASAP	genes/proteins	http://asap.ahabs.wisc.edu/asap/home.php
